# Multiparametric chemical exchange saturation transfer MRI detects metabolic changes in breast cancer following immunotherapy

**DOI:** 10.1186/s12967-023-04451-6

**Published:** 2023-08-28

**Authors:** Emily Hoffmann, Daniel Schache, Carsten Höltke, Jens Soltwisch, Stephan Niland, Tobias Krähling, Klaus Bergander, Martin Grewer, Christiane Geyer, Linda Groeneweg, Johannes A. Eble, Thomas Vogl, Johannes Roth, Walter Heindel, Bastian Maus, Anne Helfen, Cornelius Faber, Moritz Wildgruber, Mirjam Gerwing, Verena Hoerr

**Affiliations:** 1 Clinic of Radiology, University of Münster, Münster, Germany; 2 Institute of Hygiene, University of Münster, Münster, Germany; 3 Institute of Physiological Chemistry and Pathobiochemistry, University of Münster, Münster, Germany; 4 Institute of Organic Chemistry, University of Münster, Münster, Germany; 5 Institute of Immunology, University of Münster, Münster, Germany; 6 Department of Radiology, University Hospital, LMU Munich, Munich, Germany; 7 Heart Center Bonn, Department of Internal Medicine II, University Hospital Bonn, Bonn, Germany

**Keywords:** CEST, Metabolism, Tumor malignancy, Immune checkpoint inhibition, MRI

## Abstract

**Background:**

With metabolic alterations of the tumor microenvironment (TME) contributing to cancer progression, metastatic spread and response to targeted therapies, non-invasive and repetitive imaging of tumor metabolism is of major importance. The purpose of this study was to investigate whether multiparametric chemical exchange saturation transfer magnetic resonance imaging (CEST-MRI) allows to detect differences in the metabolic profiles of the TME in murine breast cancer models with divergent degrees of malignancy and to assess their response to immunotherapy.

**Methods:**

Tumor characteristics of highly malignant 4T1 and low malignant 67NR murine breast cancer models were investigated, and their changes during tumor progression and immune checkpoint inhibitor (ICI) treatment were evaluated. For simultaneous analysis of different metabolites, multiparametric CEST-MRI with calculation of asymmetric magnetization transfer ratio (MTR_asym_) at 1.2 to 2.0 ppm for glucose-weighted, 2.0 ppm for creatine-weighted and 3.2 to 3.6 ppm for amide proton transfer- (APT-) weighted CEST contrast was conducted. Ex vivo validation of MRI results was achieved by ^1^H nuclear magnetic resonance spectroscopy, matrix-assisted laser desorption/ionization mass spectrometry imaging with laser postionization and immunohistochemistry.

**Results:**

During tumor progression, the two tumor models showed divergent trends for all examined CEST contrasts: While glucose- and APT-weighted CEST contrast decreased and creatine-weighted CEST contrast increased over time in the 4T1 model, 67NR tumors exhibited increased glucose- and APT-weighted CEST contrast during disease progression, accompanied by decreased creatine-weighted CEST contrast. Already three days after treatment initiation, CEST contrasts captured response to ICI therapy in both tumor models.

**Conclusion:**

Multiparametric CEST-MRI enables non-invasive assessment of metabolic signatures of the TME, allowing both for estimation of the degree of tumor malignancy and for assessment of early response to immune checkpoint inhibition.

**Supplementary Information:**

The online version contains supplementary material available at 10.1186/s12967-023-04451-6.

## Background

The tumor microenvironment (TME) is a complex and dynamic network comprised of several cellular and non-cellular components, including a variable composition of tumor-associated immune cells. It has a significant impact on different stages of cancer progression and plays a pivotal role in therapy response [1, 2]. Since, the complexity of the TME can not be sufficiently captured with established morphological imaging techniques, functional imaging approaches that assess distinct characteristics of the TME are being developed [3]. With metabolic reprogramming being identified as a hallmark of cancer, and the continuous metabolic crosstalk between the different intratumoral cell populations contributing to cancer progression and metastasis, one main target of functional imaging is tumor metabolism [4]. In current clinical practice, tumor metabolism is investigated by positron emission tomography (PET), mainly evaluating the glycolytic activity of tumor lesions with the radiotracer ^18^F-fluorodeoxyglucose (FDG) [5]. In addition to the detection and initial staging of tumors, also treatment response is frequently assessed by repeated PET examinations. Especially immunotherapy can cause excessive infiltration of immune cells into the tumor lesion with consecutive intratumoral edema and hemorrhage [6]. These changes frequently occur without an immediate effect on lesion size, or even cause a transient increase in size [3, 7].

Even though PET imaging is an important pillar in the diagnosis, staging and follow-up of various cancer types and the spectrum of PET radiotracers is constantly increasing, it is still accompanied by several drawbacks, including radiation exposure, limited sensitivity and poor spatial resolution. Therefore, endogenous contrast mechanisms are investigated as alternatives, with rising interest in non-invasive chemical exchange saturation transfer magnetic resonance imaging (CEST-MRI). CEST is a contrast method that enables detection of a variety of low concentrated endogenous metabolites with high sensitivity and spatial resolution [8, 9]. The effect is based on selective saturation of exchangeable solute protons, bound to e.g. amine, amide, carboxyl or hydroxyl groups, by application of radiofrequency pulses at several frequency offsets. Whenever the applied frequency matches the resonance frequency of a labile proton pool, their magnetization gets saturated. Subsequently, the magnetic saturation is transferred to (unsaturated) water protons via chemical exchange and a decrease in water signal is detectable [8]. Besides the detection of intratumoral glucose, CEST-MRI additionally enables the detection of other metabolites, especially creatine and amide protons (amide proton transfer, APT). While intratumoral levels of creatine were recently found to correlate with tumor aggressiveness [10–12], amide protons are currently evaluated as markers for protein components and proliferative activity [13]. Still, previous studies have mainly focused on single metabolites, providing only limited insight into the complex interplay within the TME. To this end, we propose a multiparametric CEST-MRI approach that enables a more comprehensive insight into metabolic alterations within the TME.

This study aimed to analyze the metabolic characteristics of two syngeneic murine breast cancer models, evaluating whether the metabolic signatures assessed by multiparametric CEST-MRI allow for discrimination between the microenvironment of tumors with divergent degrees of malignancy. Furthermore, tumor-bearing mice were treated with immune checkpoint inhibitors to assess if early detection of therapeutic response is feasible by non-invasive investigation of metabolic modulations within the TME.

## Materials and methods

### Experimental outline

**Fig. 1:**
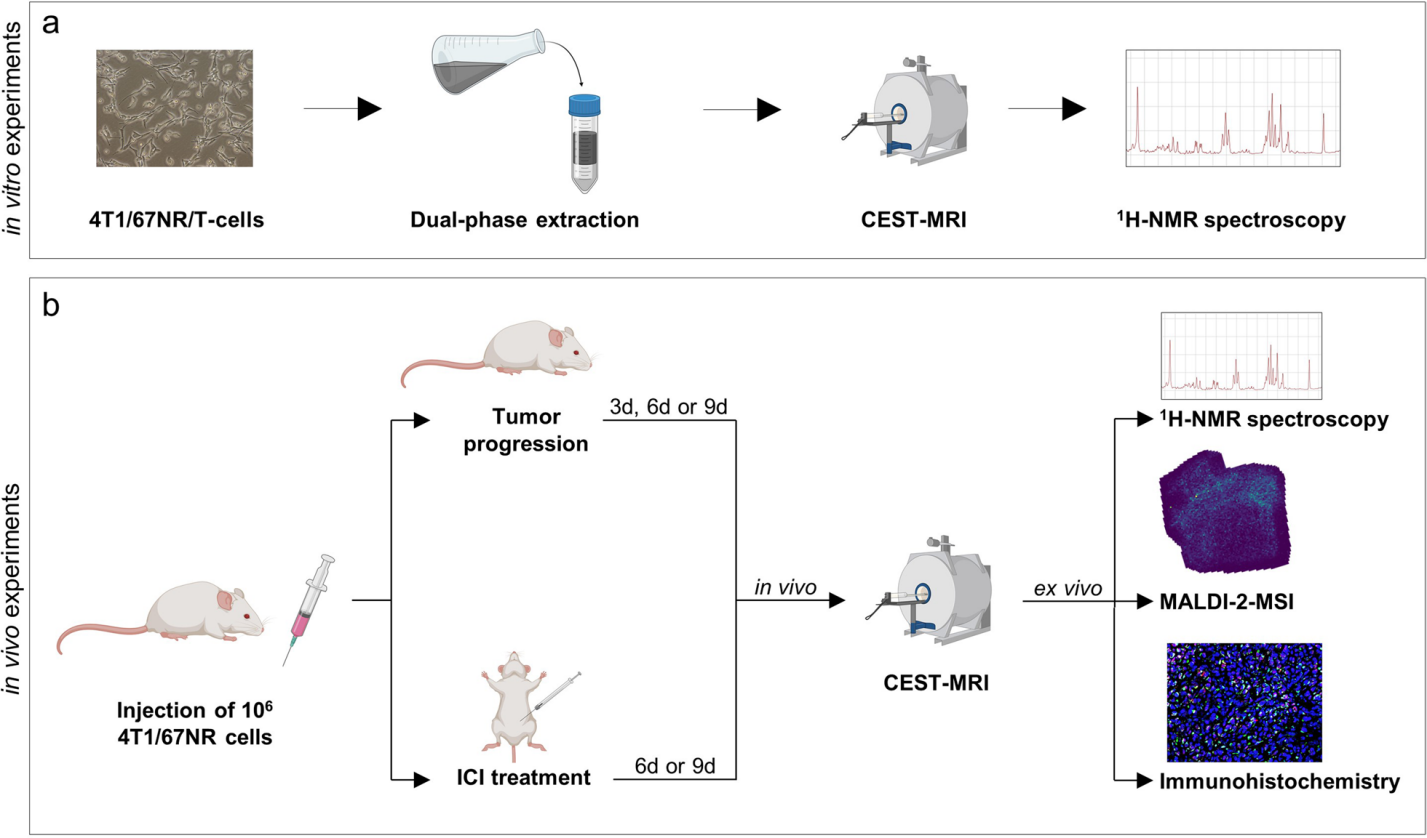
Experimental outline. **a** In vitro experiments. Water-soluble metabolites of either 4T1 or 67NR cancer cells or T-cells were extracted using a dual-phase extraction method. The metabolic profiles of the different cell types were analyzed by CEST-MRI and afterwards, results were validated by quantitative ^1^H-NMR spectroscopy. **b** In vivo experiments. 10^6^ 4T1 or 67NR cancer cells were implanted into the mammary fat pad of BALB/c mice. Longitudinal CEST-MR imaging during tumor progression was conducted on either day three, six or nine after tumor implantation. On a separate set of animals, 4T1 or 67NR tumor-bearing mice were treated with immune checkpoint inhibitors (ICI), starting three days after tumor implantation, followed by CEST-MRI on either day 6 or day 9. After the MRI scans, mice were sacrificed and ex vivo analysis of tumors was performed, including quantitative ^1^H-NMR spectroscopy, quantitative MALDI-2-MSI and immunohistochemistry. *Created using Biorender.com*

Since the malignant potential of cancer cells is driven by reprogramming of tumor metabolism, multiparametric CEST-MRI of in vitro cell extracts of either highly malignant 4T1 or low malignant 67NR cancer cells was conducted. Because cancer cells are in a complex interaction with the TME, especially with tumor-infiltrating immune cells like T-cells, in vitro CEST-MRI was additionally performed using extracts of T-cells to investigate whether the different cell populations of the TME can be separated by their metabolic profile (Fig. 1a). Subsequently, in vivo imaging of 4T1 and 67NR tumors was performed over nine days after tumor implantation in an orthotopic mouse model, in order to investigate whether multiparametric CEST-MRI enables in vivo assessment of metabolic alterations of the TME with the possibility to monitor disease progression and differentiate between two tumor models with different grades of malignancy. Furthermore, 4T1 or 67NR tumor-bearing mice were treated with immune checkpoint inhibitors to assess if early changes in tumor metabolism can be detected as indicators of therapeutic response (Fig. 1b). CEST-MRI results were confirmed by ex vivo analysis of tumor metabolism, including quantitative ^1^H nuclear magnetic resonance spectroscopy (^1^H-NMR spectroscopy), immunohistochemistry and quantitative matrix-assisted laser desorption/ionization mass spectrometry imaging with laser postionization (MALDI-2-MSI).

### Cells

Two different cell lines (highly malignant 4T1 and low malignant 67NR murine breast cancer cells) as well as freshly isolated C57BL/6 pan T-cells were investigated. Cell culture conditions are provided in Additional file 1: Method 1.

### In vitro MRI of cell extracts

To analyze the metabolic profile of different cell types in vitro, CEST-MRI of water-soluble metabolites of either cultured 4T1, 67NR or T-cells was performed at room temperature (18 °C).

Metabolites were extracted using a dual-phase extraction method [14] as described in Additional file 1: Method 2. MR imaging of the cell extracts was performed on a 9.4 T small animal BioSpec system (Bruker BioSpin GmbH, Ettlingen, Germany), equipped with a gradient system of 0.7 T/m and a 72 mm ^1^H quadrature volume resonator, using ParaVision 6.0.1 as operating system.

CEST data were measured in the frequency range of − 5.5 to 5.5 ppm relative to water resonance at 45 evenly distributed frequency offsets (frequency resolution: 0.25 ppm) using a modified 2D CEST Rapid Acquisition with Relaxation Enhancement (RARE) sequence. For saturation, one block pulse with a length of 4000 ms and a B_1_ value of 5.9 µT was used (for parameter validation, see Additional file 1: Fig. S1). Additional S_0_ data, representing the signal intensity without any saturation, were collected at ± 15 ppm for normalization. The CEST acquisition parameters were as follows: TR = 5000 ms, TE = 6.4 ms, effective TE = 38.4 ms, averages = 4, slice thickness = 1 mm, FOV = 28 × 28 mm^2^, matrix = 140 × 140, RARE factor = 12 and scan time = 2:45 h:min. For each cell type, *n* = 3 independent samples were measured. After MR imaging, the diluted in vitro cell extract samples were lyophilized for the subsequent quantitative ^1^H-NMR spectroscopy measurements.

### In vitro CEST-MRI data analysis

In vitro CEST data were analyzed using a custom-written script in Matlab (Matlab R2018a, The MathWorks, Natick, Massachusetts, USA). First, a region of interest (ROI) was drawn in the axial view covering the entire sample on a single slice. For each acquired frequency offset the signal values from pixels inside the ROI were averaged and normalized by the mean signal value of the S_0_ data to calculate a CEST spectrum. The CEST spectrum was B_0_-corrected by spline interpolation (4401 points) to correct for B_0_-inhomogeneities. Finally, magnetization transfer ratio (MTR_asym_) contrast was calculated according to $MT{R}_{asym}(\Delta \omega )=\frac{S\left(-\Delta \omega \right)-S(\Delta \omega )}{{S}_{0}}$ [15] with $S\left(\Delta \omega \right)$ being the detected signal at a frequency offset $\Delta \omega$ relative to water resonance and quantified based on either single offset values (at 2 ppm for 2 ppm peak) [16] or integration over specific frequency ranges (at 1.2–2.0 ppm for glucose [17, 18] and at 3.2–3.6 ppm for APT [19–21]). The MTR_asym_ values were normalized to the integration interval and the number of cells.

### Mouse models

Animal husbandry and experiments were carried out according to local animal welfare guidelines and were approved by responsible authorities (Landesamt für Natur, Umwelt und Verbraucherschutz NRW, Protocol No. 81-02.04.2018.A010). Female BALB/c mice (Charles River Laboratories, Sulzberg, Germany) were used at the age of eight to twelve weeks. Mice were housed under a 12 h light–dark-cycle with ad libitum access to food and water. Two syngeneic murine breast cancer models (4T1 and 67NR) were evaluated in this study. While highly malignant 4T1 tumors metastasize in regional lymph nodes and distant organs, including the lung, liver, and bones, 67NR tumors grow non-invasively and do not develop metastases [22]. For tumor implantation, 10^6^ 4T1 or 67NR cells, resuspended in 25 µL cell culture medium, were implanted orthotopically into the lower left mammary fat pad. Longitudinal MR imaging during tumor progression was conducted on either day three, six or nine after tumor implantation. In addition, separate 4T1 or 67NR tumor-bearing mice were treated with immune checkpoint inhibitors. With combination treatment enhancing the effectiveness of anticancer activity, especially in 4T1 tumors [23], immune checkpoint blockade was induced by a combination of anti-CTLA4 and anti-PD1 inhibition (BioXCell, Lebanon, USA) via i.p. injection (10 mg/kg) every second day, starting on day three after tumor inoculation [24]. MR imaging of ICI-treated mice was conducted on either day six or day nine after tumor implantation. After the scans, mice were sacrificed and tumors prepared for ex vivo experiments (Additional file 1: Method 3).

### In vivo MRI

In vivo CEST-MRI was performed on the same 9.4 T BioSpec system, using a 72 mm ^1^H quadrature volume resonator for signal excitation and a 20 mm surface coil for signal reception (Bruker BioSpin GmbH). Mice were anesthetized with 1–1.5% isoflurane in 1 L/min of oxygen and compressed air (1:4) under continuous respiratory monitoring. Animals were put on a warming bed supplied with warm water (Haake SC150, Thermo Scientific) to keep a constant body temperature of 37 °C, monitored using a rectal temperature probe. Mice were placed in supine position. To reduce susceptibility artefacts, the space between surface coil and tumor was covered with alginate (Johannes Weithas, Lütjenburg, Germany). For anatomical information, T2-weighted RARE images with TR = 2500 ms, TE = 11 ms, effective TE = 55 ms, averages = 2, slice thickness = 1 mm, slices = 12, FOV = 20 × 20 mm^2^, matrix = 256 × 256, RARE factor = 12 were acquired in sagittal and coronal plane. T2-weighted images were used to evaluate macroscopic changes after therapy (e.g. intratumoral fluid distribution) and enabled calculation of tumor volumes, analyzed using 3D Slicer (version 4.11.2021) [25].

CEST spectra were acquired in eight neighboring slices that covered the whole tumor tissue using a modified multi-slice 2D CEST RARE sequence. Thereby, data were measured in the frequency range of − 5 to 5 ppm relative to water resonance at 33 evenly distributed frequency offsets (frequency resolution: 0.3125 ppm). For saturation, one block pulse with a length of 2500 ms and a B_1_ value of 1.6 μT was used. Additional S_0_ data for normalization were collected at ± 15 ppm. The CEST acquisition parameters were as follows: TR = 3000 ms, TE = 4.7 ms, effective TE = 56.7 ms, averages = 1, slice thickness = 1 mm, FOV = 20 × 20 × 8 mm^3^, matrix = 96 × 96 × 8, RARE factor = 24 and scan time = 52:48 min:s. To analyze the intratumoral glucose uptake, CEST data acquisition was done before and after i.p. injection of glucose. To achieve a steady blood glucose level of approximately 300 mg/dL, two 100 μL boluses (1 M and 1.5 M glucose solution) were injected, separated by a 25 min break, and a post infusion spectrum was acquired five minutes after the second infusion.

After CEST imaging, dynamic contrast-enhanced (DCE) MRI using Magnevist (Gd-DTPA, 0.3 mmol/kg) was performed to exclude that glucose-weighted CEST results are dominated by perfusion effects (Additional file 1: Method 7).

### In vivo CEST-MRI data analysis

CEST data of in vivo imaging were analyzed by a different custom-written script in Matlab (MATLAB R2018a, The MathWorks).

CEST data were evaluated pixel by pixel for each slice that contained tumor tissue. Thereby, a ROI was drawn covering the whole tumor in the respective slice and the following analysis steps were performed: Signal values from each pixel were normalized by the mean value of both S_0_ measurements, resulting in an individual CEST spectrum for each pixel. After B_0_-correction by using spline interpolation (4001 points), MTR_asym_ contrast was calculated similar to in vitro evaluation and quantified pixel by pixel to calculate asymmetry maps. Next, all CEST spectra from individual pixels within the ROI were averaged in their common frequency range to calculate a slice-specific CEST spectrum. This analysis pipeline was repeated for each slice which contained tumor tissue. Finally, all slice-specific spectra were averaged based on the number of pixels in the defined ROI of the individual slice, resulting in one global CEST spectrum for each data set (Additional file 1: Fig. S2). For the global CEST spectrum, MTR_asym_ contrast was calculated similar to the in vitro study and quantified at 2 ppm (creatine), 1.2–2.0 ppm (glucose) and 3.2–3.6 ppm (APT). MTR_asym_ values were normalized to the integration interval. ∆Glc values were determined as difference of glucose-weighted CEST contrast after and before glucose infusion.

### Ex vivo experiments

Quantitative analysis of cell extracts as well as extracts of snap-frozen tumors was performed by ^1^H-NMR spectroscopy (Additional file 1: Method 4). Spectra were recorded at 600 MHz using an Agilent DD2 600 spectrometer (Agilent Technologies, Santa Clara, California, USA). In addition, for dedicated analysis of glucose uptake and the intratumoral immune cell infiltrate, a co-staining of the glucose transporters GLUT1 and GLUT3 and the T-cell marker CD3 was conducted (Additional file 1: Method 5). While exemplary ROIs are presented within the respective figures, total cross-sections of the co-stained tumor tissue are shown in Additional file 1: Figure S3. For further analysis of the intratumoral glucose accumulation, quantitative MALDI-2-MSI of tumor sections was performed using isotopically labeled standard and a novel tandem-MS based method (Additional file 1: Method 6) [26].

### Statistical analysis

Statistics were performed using GraphPad Prism (version 9.2.0, GraphPad Software Inc., San Diego, USA). Descriptive statistics and further details of the analysis of significance are provided in Additional file 1: Table S1–S6. Shapiro–Wilk test was performed to check for normal distribution of data (α = 0.05).

In vitro: Two-sided t-tests were performed to compare the normally distributed cell extracts (*n* = 3 for each cell line).

In vivo: CEST contrasts of longitudinal in vivo data were evaluated by one-way ANOVA with Tukey post-hoc test or Kruskal–Wallis test (depending on *p*-value of the respective Shapiro–Wilk test). CEST contrasts of therapy groups were compared to control groups using a two-sided t-test or a Mann–Whitney U test (depending on *p*-value of the respective Shapiro–Wilk test). Correlation analyses were performed by calculating corresponding Spearman rank correlation coefficients: between non-Gaussian distributed CEST-MRI contrasts and metabolite concentrations quantified by ^1^H-NMR spectroscopy; and between glucose-weighted CEST-MRI contrast (MTR_asym_(∆Glc)) and DCE-MRI results (K_trans_). In vivo group sizes were as follows: *n* = 6, *n* = 7, *n* = 5 for 4T1 tumors on days three, six and nine, respectively; *n* = 5, *n* = 5, *n* = 5 for 67NR tumors on days three, six and nine, respectively; *n* = 5, *n* = 5 for ICI-treated 4T1 tumors on days six and nine, respectively; *n* = 6, *n* = 5 for ICI-treated 67NR tumors on days six and nine, respectively.

## Results

### Metabolic profiles of cell extracts using CEST-MRI

The capability of CEST-MRI to assess cell type-specific metabolic profiles of different cell populations within the TME was investigated using in vitro cell extracts.

**Fig. 2:**
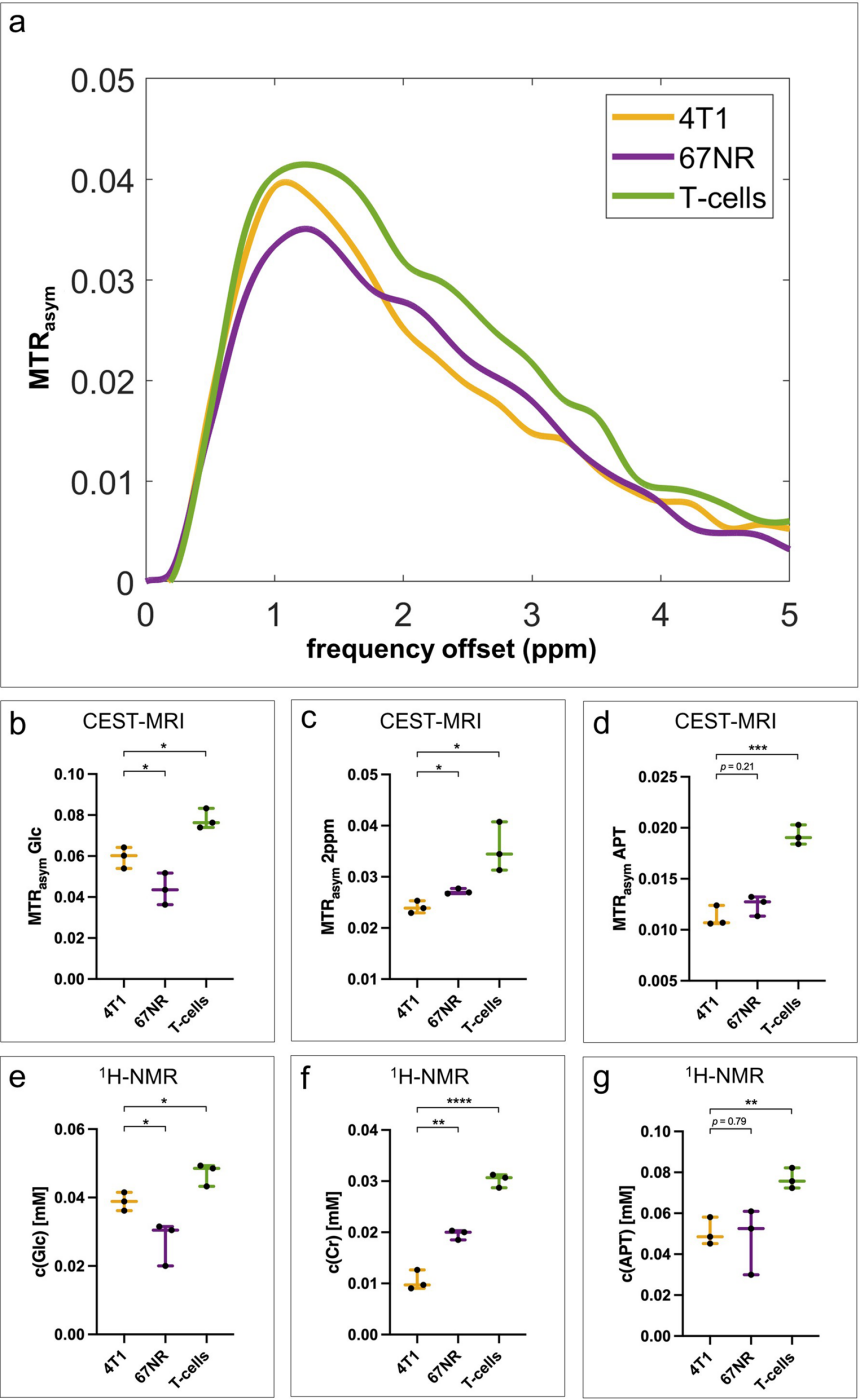
Metabolic profiles of in vitro cell extracts. **a** Exemplary CEST-MRI-derived MTR_asym_ spectra of 4T1 (yellow), 67NR (purple) or T-cell (green) extracts. **b-d** Quantitative MTR_asym_ for Glc-, 2 ppm- and APT-weighted CEST contrast of the different cell extracts. Extracts of highly malignant 4T1 breast cancer cells demonstrated higher Glc- and APT-weighted and lower 2 ppm-weighted CEST contrast compared to low malignant 67NR cell extracts. T-cell extracts showed higher CEST contrast at all three examined frequency offsets. **e–g** Metabolite concentrations (glucose (Glc), creatine (Cr), amide protons (APT)) quantified by ^1^H-NMR spectroscopy, with similar metabolic differences between the cell lines than observed with CEST-MRI. Each dot represents one sample, with horizontal lines indicating minimum, mean and maximum values of each respective group. ^*^p < 0.05, ^**^p < 0.01, ^***^p < 0.001, ^****^p < 0.0001

MTR_asym_ spectra differed between highly malignant 4T1 and low malignant 67NR murine breast cancer cells (Fig. 2a). Highly malignant 4T1 cell extracts demonstrated higher glucose-weighted CEST contrast compared to low malignant 67NR cell extracts (Fig. 2b). The CEST effect at 2 ppm mainly represents creatine [16], hereafter referred to as creatine-weighted CEST-MRI. In contrast to the glucose contrast, creatine was significantly higher in 67NR than in 4T1 cell extracts (Fig. 2c). No significant differences were found in APT-weighted CEST contrast (Fig. 2d). MTR_asym_ spectra of T-cells, as one of the main contributors of tumor-associated inflammation, differed significantly from those of the two cancer cell lines, with T-cell extracts showing higher CEST contrast for all three examined metabolites (Fig. 2a–d).

CEST-MRI results were validated by quantitative ^1^H-NMR spectroscopy that revealed similar differences between the cell types for all three analyzed metabolites (Fig. 2e–g). Exemplary ^1^H-NMR spectra are shown in Additional file 1: Figure S4.

### In vivo CEST-MRI to monitor tumor progression

To further develop CEST-MRI as a diagnostic tool for assessment of tumor malignancy and progression, longitudinal in vivo imaging was conducted and evaluated in highly malignant 4T1 and low malignant 67NR breast cancer models.

Anatomical T2-weighted imaging revealed a time-dependent increase in tumor volume from day three to day nine in both tumor models. Highly malignant 4T1 tumors grew faster than 67NR tumors and exhibited central areas of intratumoral necrosis, while 67NR tumors demonstrated homogenous morphology with only minor intratumoral necrosis, as reported previously [27]. At all time points, volumes of low malignant 67NR tumors were smaller than volumes of 4T1 tumors (Additional file 1: Table S3).

**Fig. 3:**
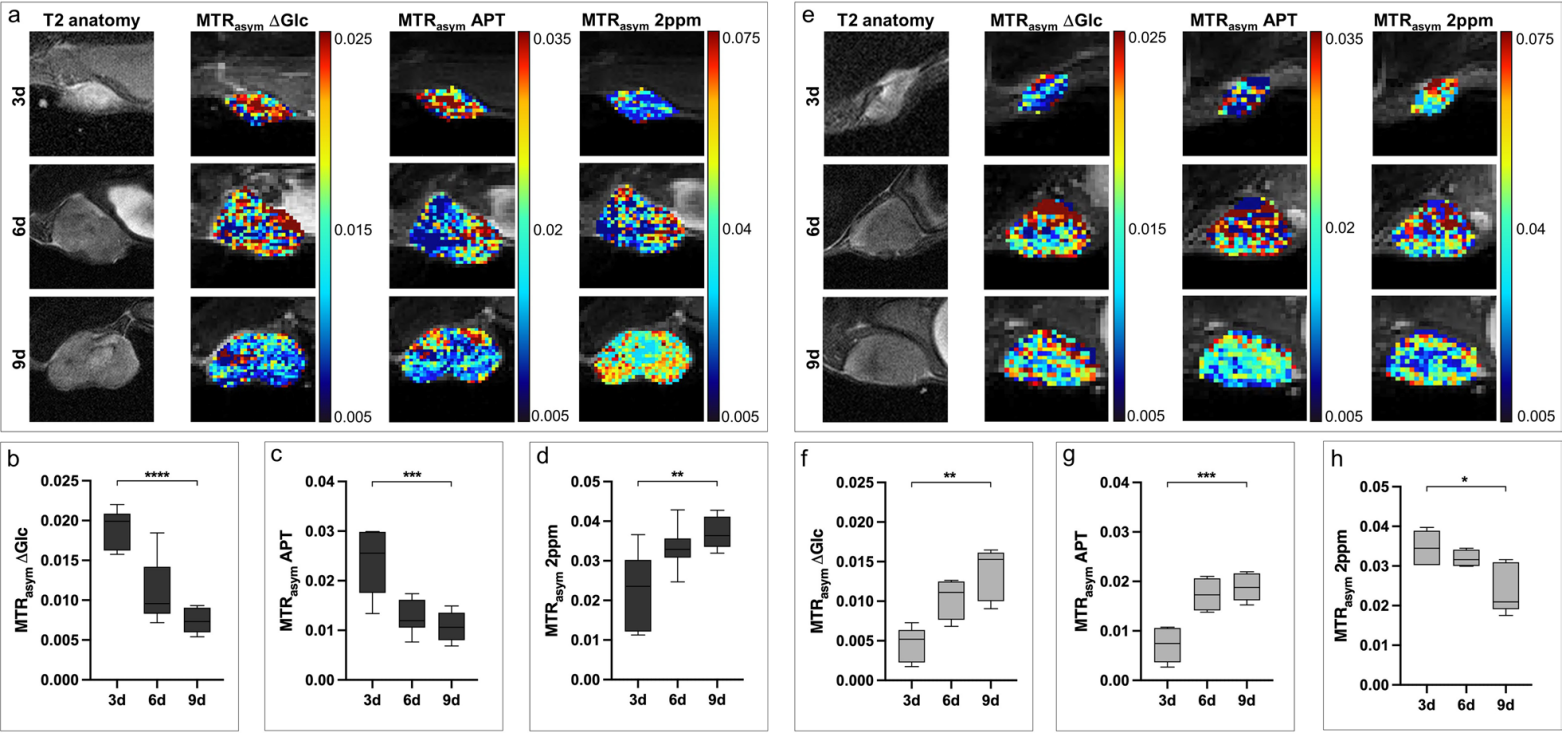
In vivo CEST-MRI during tumor progression. **a** Exemplary T2-weighted images and pixelwise MTR_asym_ maps for ∆Glc-, APT- or 2 ppm-weighted CEST contrast of 4T1 tumors on day three, six and nine after tumor implantation. **b-d** Boxplots showing quantitative MTR_asym_ for ∆Glc-, APT- and 2 ppm-weighted CEST contrast of whole 4T1 tumors on day three, six and nine after tumor implantation, with the horizontal line indicating mean and boxes representing the 10^th^ and 90^th^ percentile. During progression of 4T1 tumors, ∆Glc- and APT-weighted CEST contrast decreased, while 2 ppm-weighted CEST contrast increased from day three to day nine. **e** Exemplary T2-weighted images and pixelwise MTR_asym_ maps for ∆Glc-, APT- and 2 ppm-weighted CEST contrast of 67NR tumors on day three, six and nine after tumor implantation. **f–h** Boxplots showing quantified MTR_asym_ for ∆Glc-, APT- and 2 ppm-weighted CEST contrast of whole 67NR tumors on day three, six and nine after tumor implantation, with the horizontal line indicating the mean, and boxes representing the 10th and 90th percentile. During progression of 67NR tumors, ∆Glc- and APT-weighted CEST contrast increased, while 2 ppm-weighted CEST contrast decreased from day three to day nine. ^*^p < 0.05, ^**^p < 0.01, ^***^p < 0.001, ^****^p < 0.0001

During progression of 4T1 tumors, glucose-weighted CEST contrast significantly decreased from day three to day nine. Additionally, also APT-weighted CEST contrast decreased over time, while creatine-weighted CEST contrast increased during progression of 4T1 tumors (Fig. 3a–d). Low malignant 67NR tumors showed significantly increasing glucose-weighted CEST contrast from day three to day nine, similar to the increase in APT-weighted CEST contrast. Creatine-weighted CEST contrast decreased during progression of 67NR tumors (Fig. 3e–h).

For all three investigated metabolites, significant differences were observed between the two tumor models on day three and day nine, while on day six, only the APT-weighted CEST contrast showed substantial differences (Additional file 1: Table S3, S4). Exemplary CEST spectra are displayed in Additional file 1: Figure S5a. Only a low correlation between glucose-weighted CEST contrast and DCE-derived tumor perfusion was observed (Additional file 1: Fig. S6).

### Ex vivo analysis of tumor metabolism and cellular composition during tumor progression

To investigate the diagnostic power of multiparametric CEST-MRI, ex vivo ^1^H-NMR spectroscopy of homogenized tumor tissue as well as immunohistochemistry and MALDI-2-MSI of tumor sections were performed.

For all analyzed metabolites (glucose, amide protons and creatine), ^1^H-NMR spectroscopic data revealed a significant correlation with the quantified CEST contrast at all three time points, confirming the validity of the in vivo CEST approach (Additional file 1: Fig. S7a-c).

**Fig. 4:**
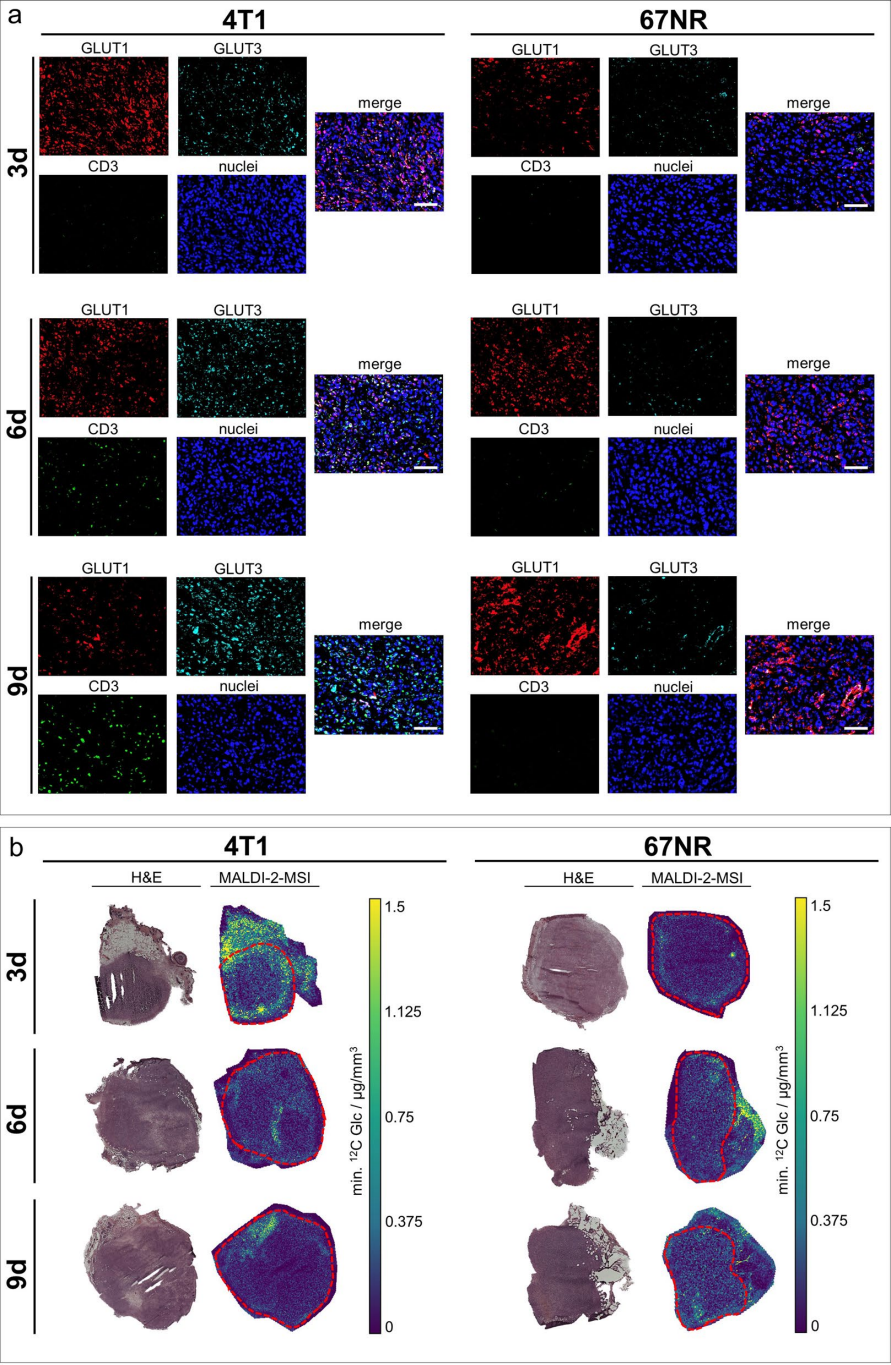
Ex vivo analysis of tumor metabolism during tumor progression. **a** Representative sections of 4T1 and 67NR tumors after 4-channel staining of GLUT1 (red), GLUT3 (turquoise), CD3 (green) and nuclei (blue) with correlating merged images. During progression of 4T1 tumors, a decrease in GLUT1, an increase in CD3 as well as an increase in GLUT3 expression were observed. In contrast, 67NR tumors showed increased GLUT1 expression from day three to day nine, with constantly low expression of GLUT3 and CD3. Scale bars indicate 100 µm. **b** Exemplary images of MALDI-2-MS with corresponding H&E stainings showing decreased intratumoral glucose accumulation during progression of 4T1 tumors, in contrast to 67NR tumors exhibiting increased glucose accumulation over time. Tumor contours are marked with dashed red lines

As the creatine level was strikingly high in late-stage 4T1 tumors and was already identified as a major contributor in T-cell metabolism (Fig. 2), immunohistochemical analysis of the intratumoral immune cell infiltrate was performed. Increased expression of CD3, a pan T-cell marker, was observed during progression of 4T1 tumors, compared to constantly low expression of CD3 in 67NR tumors (Fig. 4a).

Specifically for the validation of glucose-weighted CEST results and the intracellular glucose uptake, additional immunohistochemical stainings of the glucose transporters GLUT1 and GLUT3 were applied and evaluated visually. While GLUT1 is associated with the overall intratumoral glucose uptake, overexpression of GLUT3 is known to promote metastasis of triple negative breast cancer [28]. Over time, a decrease of GLUT1 was observed in 4T1 tumors, while GLUT1 expression increased in the 67NR model, indicating an increase in glucose demand and metabolic turnover. GLUT3 was highly expressed in 4T1 tumors, increasing from day three to day nine; contrary to that, 67NR tumors showed constantly low expression of GLUT3 (Fig. 4a). MALDI-2-MSI showed decreasing intratumoral glucose concentrations over time in 4T1 tumors, while it confirmed increasing intratumoral glucose concentrations during progression of 67NR tumors (Fig. 4b). Exemplary quantitative analysis of intratumoral glucose concentrations assessed by MALDI-2-MSI of tumor slices showed significantly higher glucose concentrations in 67NR compared to 4T1 tumors on day nine after tumor implantation (Additional file 1: Fig. S8).

### Response assessment to immune checkpoint blockade

With the aim to investigate whether multiparametric CEST-MRI is able to assess early response to immune checkpoint blockade by detecting therapy-induced metabolic changes within the TME, 4T1 or 67NR tumor-bearing mice were treated with a combination therapy of anti-PD1 and anti-CTLA4.

While volumes of 67NR tumors were significantly reduced after ICI treatment, ICI-treated 4T1 tumors showed significantly increased volumes compared to control groups (Additional file 1: Table S5).

**Fig. 5:**
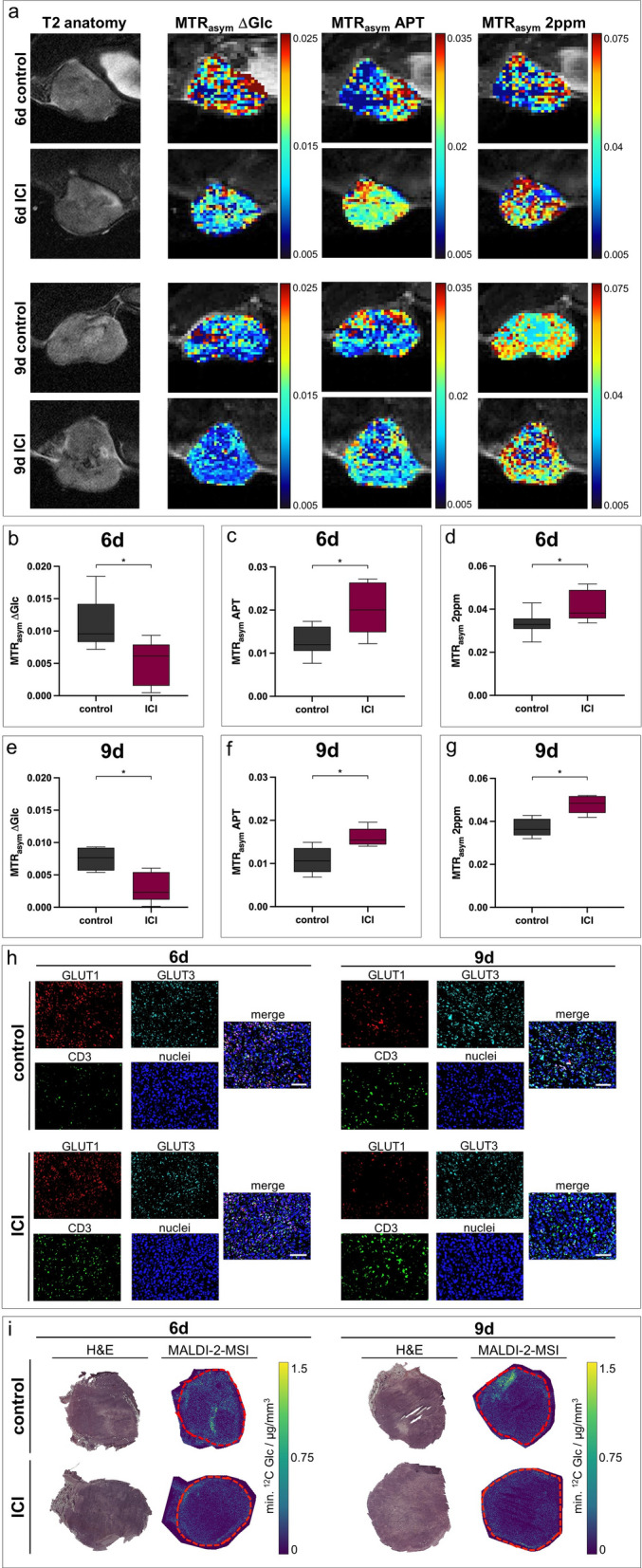
In vivo CEST-MRI of ICI-treated 4T1 tumors and respective ex vivo analysis. **a** Exemplary T2-weighted images with pixelwise MTR_asym_ maps for ∆Glc-, APT- or 2 ppm-weighted CEST contrast of ICI-treated 4T1 tumors on day six and day nine compared to untreated controls. **b-g** Boxplots showing quantified MTR_asym_ for ∆Glc-, APT- and 2 ppm-weighted CEST contrast of whole tumors, with the horizontal line indicating the mean, and boxes representing the 10th and 90th percentile. After ICI treatment, ∆Glc-weighted CEST contrast decreased, while APT- and 2 ppm-weighted CEST contrast increased after treatment on day six (**b**–**d**) and day nine (**e**–**g**). ^*^p < 0.05. **h** Exemplary sections of 4T1 tumors after 4-channel staining of GLUT1 (red), GLUT3 (turquoise), CD3 (green) and nuclei (blue) with correlating merged images. ICI-treated tumors showed increased CD3 expression. GLUT1 expression decreased after treatment, while expression of GLUT3 was approximately constant. Scale bars indicate 100 µm. **i** Exemplary images of MALDI-2-MS with corresponding H&E stainings showing reduced intratumoral glucose accumulation after ICI therapy on both day 6 and day 9. Tumor contours are marked with dashed red lines

After ICI therapy, 4T1 tumors exhibited decreased glucose-weighted CEST contrast on day six and day nine. However, both creatine- and APT-weighted CEST contrast increased after treatment compared to control groups (Fig. 5a–g) (Additional file 1: Table S5-S6). Exemplary CEST spectra are shown in Additional file 1: Figure S5b.

Ex vivo analysis clearly confirmed the in vivo CEST-MRI results post-therapy; the elevated creatine level was in concordance with increased intratumoral CD3 expression, assessed by immunohistochemistry. Furthermore, immunohistochemical staining showed decreased GLUT1 and approximately constant GLUT3 expression, in line with MALDI-2-MSI validating decreased intratumoral glucose concentrations after ICI therapy (Fig. 5h, i).

**Fig. 6:**
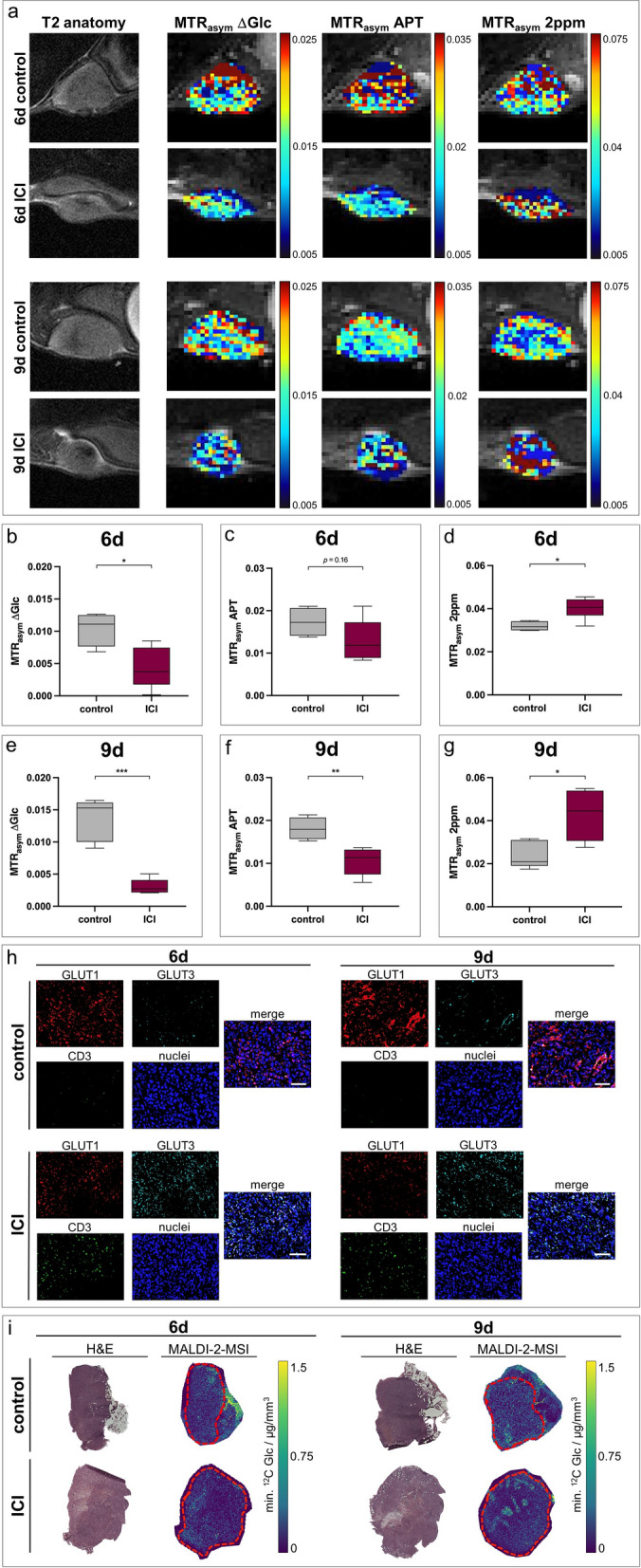
In vivo CEST-MRI of ICI-treated 67NR tumors and respective ex vivo analysis. **a** Exemplary T2-weighted images with pixelwise MTR_asym_ maps for ∆Glc-, APT- or 2 ppm-weighted CEST contrast of ICI-treated 67NR tumors on day six and day nine compared to untreated controls. **b-g** Boxplots showing quantified MTR_asym_ for ∆Glc-, APT- and 2 ppm-weighted CEST contrast of whole tumors, with the horizontal line indicating the mean, and boxes representing the 10th and 90th percentile. After ICI treatment, ∆Glc-weighted and 2 ppm-weighted CEST contrast increased after treatment on day six (**b**–**d**) and day nine (**e**–**g**). In contrast, APT-weighted CEST contrast decreased after treatment. ^*^p < 0.05. **h** Exemplary sections of 67NR tumors after 4-channel staining of GLUT1 (red), GLUT3 (turquoise), CD3 (green) and nuclei (blue) with correlating merged images. ICI-treated tumors showed excessively increased CD3 expression. GLUT1 expression decreased after treatment, while GLUT3 expression increased. Scale bars indicate 100 µm. **i** Exemplary images of MALDI-2-MS with corresponding H&E stainings showing reduced intratumoral glucose accumulation after ICI therapy on both day six and day nine. Tumor contours are marked with dashed red lines

Similar to the 4T1 model, ICI-treated 67NR tumors showed decreased glucose-weighted CEST contrast compared to control groups, accompanied by increased creatine-weighted CEST contrast. However, contrary to the 4T1 model, 67NR tumors exhibited decreased APT-weighted CEST contrast after ICI treatment (Fig. 6a–g). Intratumoral CD3 expression was raised after therapy on day six and day nine, similar to increased GLUT3 expression, while GLUT1 expression was decreased (Fig. 6h). MALDI-2-MSI confirmed glucose-weighted CEST results by showing reduced intratumoral glucose concentrations of 67NR tumors after treatment (Fig. 6i).

Quantitative validation of CEST-MRI results for both tumor models was performed by ^1^H-NMR spectroscopy of homogenized tumor tissue. Spearman correlation analysis of CEST-MRI contrast and metabolite concentrations quantified with ^1^H-NMR spectroscopy showed a significant correlation for all analyzed metabolites (Additional file 1: Fig. S7d–f).

## Discussion

This study demonstrates the capability of multiparametric CEST-MRI for non-invasive assessment of metabolic profiles of murine cancer models, enabling characterization of their malignant potential as well as early response to immunotherapy.

Our in vitro results showed that cell type-specific metabolic profiles of cell extracts could be detected by all three CEST contrasts investigated: glucose, creatine and APT. These enabled to discriminate between highly and low malignant cancer cells (4T1, 67NR) and immune cells (T-cells). The increased glucose-weighted CEST contrast in 4T1 cell extracts reflected their higher glycolytic activity compared to 67NR cells, associated with their greater malignant potential [29]. Additionally, both cancer cell lines differed significantly in the calculated CEST contrast at a frequency offset of 2 ppm, which is closely related to their creatine metabolism, showing a higher creatine level in low malignant 67NR compared to highly malignant 4T1 cells. While these results are consistent with first clinical studies that observed an inverse correlation between creatine metabolism and cancer aggressiveness [10], this has not been previously investigated in cancer cells in vitro. The metabolic profile of T-cells, calculated from CEST-MRI, clearly differed from both cancer cell lines, inter alia capturing high creatine levels, which were recently found to be a specific characteristic of this immune cell population [12]. Thus, multiparametric CEST-MRI appears to be a promising tool to distinguish between different populations of the TME, and their degree of malignancy, based on their metabolic profiles.

This perspective could be confirmed in subsequent in vivo experiments. CEST contrasts of all three investigated metabolites showed significant changes during tumor progression from day three to day nine, with strongly divergent trends between both tumor models. At early stage on day three, the intratumoral glucose metabolism was dominated by the respective characteristics of the different cancer cells, with higher glycolytic activity of 4T1 cells as shown in the in vitro experiments. Subsequent intratumoral changes might lead to different properties of glucose metabolism at later time points. Rapid growth of 4T1 tumors led to increased necrosis and decreased metabolic activity of the tumor, whereas increased relative growth of 67NR tumors [30] was in line with an increase in proliferative activity and pronounced glucose metabolism. These changes in glucose metabolism and the proportions of glucose uptake between 4T1 and 67NR tumors, assessed by non-invasive CEST-MRI, were in concordance with a previous ^18^F-FDG-PET study of these tumor models [29].

Changes of APT-weighted CEST contrast showed similar trends as glucose-weighted CEST contrast. High APT-weighted CEST contrast was observed for 4T1 tumors on day three with a consecutive strong decrease until day nine, while APT-weighted CEST contrast of 67NR tumors increased over time. In a first clinical study on endometrial adenocarcinomas, a positive correlation between APT-weighted CEST contrast and the histologically determined grade of tumor malignancy was observed [31]. In our preclinical study, this correlation could only be confirmed for day three; later, both breast cancer models showed opposing trends. As APT-weighted CEST contrast is also considered as marker for protein components and the proliferative activity of tumors [32], the observed changes from day three to day nine in 4T1 tumors can be attributed to increasing tumor necrosis, markedly in contrast to 67NR tumors.

In malignant diseases of the central nervous system, tumor progression is associated with a decrease in creatine metabolism [10, 16]. While changes in creatine metabolism of low malignant 67NR breast tumors were in line with these results, highly malignant 4T1 tumors showed increased creatine metabolism during progression. These results were accompanied by excessive infiltration of T-cells into the TME of 4T1 tumors on day six and day nine, suggesting that metabolic changes detected by CEST-MRI might be able to detect alterations of the intratumoral immune cell infiltrate. Since this study only analyzed the metabolic changes of 4T1 and 67NR tumors, representing an extreme manifestation of the different grades of malignancy (4T1: highly malignant, 67NR: low malignant), further investigations of intermediate-grade tumors should certainly be a goal for future studies.

CEST contrasts were able to serve as early biomarkers for therapeutic response in targeted therapy. Already three days after initiation of ICI treatment, glucose-weighted CEST contrast decreased and creatine metabolism increased in both tumor models. This is significantly earlier than in a study evaluating treatment response to ICI with ^18^F-FDG-PET in a mouse model of hepatocellular carcinoma, with the first significant changes in metabolism observed after fourteen days [33]. While a decrease in glucose metabolism, assessed by CEST-MRI, has also been observed as sign of successful response to chemotherapy [34], the increase in creatine metabolism detected by CEST-MRI appears to be attributable to the pronounced intratumoral T-cell infiltration after ICI therapy, a specific hallmark for successful anticancer immunotherapy [11]. The effects of ICI therapy on amide proton metabolism showed clear differences between the two tumor models: ICI-treated 4T1 tumors demonstrated elevated amide proton metabolism compared to control groups, whereas 67NR tumors showed decreased values after therapy. Since 4T1 and 67NR tumors differ in their PD-L1 expression [35], these differences might potentially indicate different activation patterns of the intratumoral T-cells; however, further investigation of this hypothesis is needed. Previous CEST-MRI studies were also able to detect changes in individual metabolites after different cancer therapies [34, 36, 37]. However, these changes of tumor metabolism have mainly been shown after size-reduction of tumors due to cytostatic chemotherapeutics. The multiparametric CEST-MRI setup of this study now clearly shows that the effects of targeted tumor therapeutics, such as immunotherapeutics, can also be detected at an early stage, even before reliable changes in tumor volumetry occur.

Despite the demonstrated strength of the proposed multiparametric CEST approach, quantitative CEST results have to be interpreted carefully. Especially the detected changes in glucose levels might be affected by perfusion-related properties, as some glycolytic intermediates are also sensitive to the CEST contrast. Here, only a low correlation between glucose-weighted CEST contrast and the DCE-derived parameter K_trans_ was observed, indicating that CEST-MRI results are predominantly determined by metabolic activity. In general, CEST-MRI is sensitive to multiple metabolites with labile protons. Especially in vivo CEST-MRI spectra contain contributions from various exchangeable proton groups, and are further influenced by physio-chemical effects like nuclear Overhauser effects or conventional magnetization transfer. Thereby their individual chemical exchange rate depends on metabolic concentrations and is influenced by pH, temperature and the chemical environment [38]. Since 4T1 and 67NR tumors differ in these tumor characteristics, for example 4T1 tumors potentially having slightly lower pH than 67NR tumors [39], the differences in tumor metabolism between the different experimental groups could have been additionally magnified by these effects. As the frequency offset at which the maximum CEST signal can be detected depends on local concentration, pH and temperature as well, quantification based on a single MTR_asym_ value, such as creatine at a frequency offset of 2 ppm, may reduce quantification accuracy. Therefore, a quantification approach based on the integration over a frequency range (in this study: glucose-weighted CEST contrast at 1.2–2.0 ppm, APT-weighted CEST contrast at 3.2–3.6 ppm) rather than on a single offset could lead to more reproducible results, even though it may result in reduced specificity because of contaminating upfield and downfield CEST contributions. As an example, prominent nuclear Overhauser effects (NOEs) were reported at -1.6 ppm, mainly originating from the choline head group of phospholipids, and at -3.5 ppm, related to macromolecular components such as mobile proteins and lipids [40, 41]. As phospholipid metabolism is associated with both tumor progression and response to cancer therapies [42, 43], it could affect the quantified CEST contrast in addition to other metabolic changes. Although other quantification approaches such as multi-pool fitting techniques [48, 49] are currently subject of research to overcome these limitations in accuracy of CEST results, calculation of the asymmetry is still the gold-standard technique of quantification. Considering these limitations in the quantification accuracy, the metabolic alterations detected by CEST-MRI were thoroughly validated by several quantitative or semi-quantitative ex vivo analysis techniques, providing evidence that the calculated CEST-MRI parameters reliably capture the characteristics of tumor metabolism. However, in contrast to a spatially resolved analysis of glucose using MALDI-2-MSI, creatine and amide protons were only analyzed by ^1^H-NMR spectroscopy after extracting the analytes from tissue, prohibiting any spatial information.

Finally, both for diagnostic and therapeutic follow-up, the translation of our multiparametric CEST-MRI technique into the clinical setting is yet challenging: At lower field strengths, the amplitude of the CEST effect is substantially reduced due to lower net magnetization and shortened T_1_ relaxation time, which can not be fully compensated by applying saturation pulses with long durations or larger amplitudes (higher B_1_ values) without considering a high specific absorption rate [44]. Moreover, the worse frequency separation of CEST effects at lower field strengths reduces CEST sensitivity and specificity. To overcome these limitations, various approaches have been performed to optimize sequence and pulse design at clinical field strength, or to improve CEST contrast by postprocessing techniques [45].

## Conclusions

This study demonstrates that metabolic signatures derived by non-invasive multiparametric CEST-MRI enable a clear discrimination between tumors with divergent degrees of malignancy. Furthermore, based on changes of these metabolic signatures, it allows for early response assessment to immune checkpoint inhibition, even before a decrease in lesion size occurs.

### Supplementary Information

**Additional file 1. **Additional methods 1–7, Figures S1–S8 and Tables S1–S6.

## Data Availability

The datasets generated during and/or analyzed during the current study are available from the corresponding author on reasonable request.

## Abbreviations

APT: Amide proton transfer
CEST: Chemical exchange saturation transfer
DCE: Dynamic contrast-enhanced
FDG: Fluorodeoxyglucose
FOV: Field of view
Glc: Glucose
ICI: Immune checkpoint inhibitor
K_trans_: Volume transfer constant
MALDI-MSI: Matrix-assisted laser desorption/ionization mass spectrometry imaging
MRI: Magnetic resonance imaging
MTR_asym_: Asymmetric magnetization transfer ratio
NMR: Nuclear magnetic resonance
PET: Positron emission tomography
RARE: Rapid acquisition with relaxation enhancement
ROI: Region of interest
TE: Echo time
TME: Tumor microenvironment
TR: Repetition time
