# Supplementary material for: Multiparametric chemical exchange saturation transfer MRI detects metabolic changes in breast cancer following immunotherapy
Source: J Transl Med. 2023 Aug 28;21:577. doi: 10.1186/s12967-023-04451-6 (PMC10463706; doi:10.1186/s12967-023-04451-6)
Supplement: Supplementary file 1 — Additional file 1. Additional methods 1–7, Figures S1–S8 and Tables S1–S6. [file 12967_2023_4451_MOESM1_ESM.pdf]

Multiparametric chemical exchange saturation transfer MRI detects metabolic changes in breast cancer following immunotherapy – Supplementary Material

Emily Hoffmann[1,*,#], Daniel Schache[1,*], Carsten Höltke[1], Jens Soltwisch[2], Stephan Niland[3], Tobias Krähling[1], Klaus Bergander[4], Martin Grewer[1], Christiane Geyer[1], Linda Groeneweg[5], Johannes A. Eble[3], Thomas Vogl[5], Johannes Roth[5], Walter Heindel[1], Bastian Maus[1], Anne Helfen[1], Cornelius Faber[1], Moritz Wildgruber[1,6], Mirjam Gerwing[1,†], Verena Hoerr[1,7,†]

[1]University of Münster, Clinic of Radiology, Münster, Germany
[2]University of Münster, Institute of Hygiene, Münster, Germany
[3]University of Münster, Institute of Physiological Chemistry and Pathobiochemistry, Münster, Germany
[4]University of Münster, Institute of Organic Chemistry, Münster, Germany
[5]University of Münster, Institute of Immunology, Münster, Germany
[6]Department of Radiology, University Hospital, LMU Munich, Munich, Germany
[7]Heart Center Bonn, Department of Internal Medicine II, University Hospital Bonn, Bonn, Germany

[*,†]These authors contributed equally.
[#]Correspondence: emily.hoffmann@ukmuenster.de

Table of content

Suppl. Method 1	Cell culture conditions.
Suppl. Method 2	Dual-phase extraction of water-soluble metabolites of cultured 4T1, 67NR and T-cells.
Suppl. Method 3	Preparation of tumors for *ex vivo* analysis.
Suppl. Method 4	^{1}H-NMR spectroscopy.
Suppl. Method 5	Immunohistochemical co-staining of GLUT1, GLUT3 and CD3.
Suppl. Method 6	Matrix-assisted laser desorption/ionization mass spectrometry imaging (MALDI-2-MSI).
Suppl. Method 7	Dynamic contrast-enhanced MRI (DCE-MRI).
Suppl. Figure 1	Exemplary CEST spectra of *in vitro* cell extracts for different B_1 values.
Suppl. Figure 2	Pipeline for *in vivo* CEST-MRI data analysis.
Suppl. Figure 3	Complete cross-sections of GLUT1, GLUT3 and CD3 co-stained tumors.
Suppl. Figure 4	Exemplary ^{1}H-NMR spectra of *in vitro* cell extracts.
Suppl. Figure 5	Exemplary CEST spectra of *in vivo* CEST-MRI.
Suppl. Figure 6	Correlation between *in vivo* glucose-weighted CEST-MRI and DCE-MRI.
Suppl. Figure 7	Correlation between *in vivo* CEST-MRI and *ex vivo* ^{1}H-NMR spectroscopy.
Suppl. Figure 8	Exemplary comparison of intratumoral ^{12}C glucose concentrations assessed by MALDI-2-MSI.
Suppl. Table 1	Descriptive statistics of *in vitro* cell extracts.
Suppl. Table 2	Analysis of statistical significance of *in vitro* cell extracts.
Suppl. Table 3	Descriptive statistics of longitudinal *in vivo* study.
Suppl. Table 4	Analysis of statistical significance of longitudinal *in vivo* study.
Suppl. Table 5	Descriptive statistics of *in vivo* ICI therapy.
Suppl. Table 6	Analysis of statistical significance of *in vivo* ICI therapy

Suppl. Method 1: Cell culture conditions.

4T1 and 67NR breast cancer cells were cultured in Dulbecco's modified Eagle's medium (DMEM, Thermo Fisher Scientific, Waltham, Massachusetts, USA), supplemented with 10% fetal bovine serum. T-cells were isolated from spleens of C57BL/6 mice as previously published [1]. Isolated T-cells were cultured in RPMI 1640 medium (Sigma-Aldrich, Burlington, USA), supplemented with 10% fetal bovine serum, 1% penicillin (10 kU/mL) / streptomycin (10 mg/mL), 1% L-glutamine, 1% sodium pyruvate and 1% hydroxyethyl piperazineethanesulfonic acid (HEPES, Thermo Fisher Scientific). Culture conditions for all cells were 37°C, 5% CO_2 and 100% humidity.

Suppl. Method 2: Dual-phase extraction of water-soluble metabolites of cultured 4T1, 67NR and T-cells.

For MR imaging of cell extracts, water-soluble metabolites of either 4T1, 67NR or T-cells were prepared using a dual-phase extraction method. Cells were cultured as monolayers until 80% confluency. After washing the cells twice with PBS, 4 mL ice-cold methanol were added. The cells were harvested and 4 mL chloroform and 4 mL water were added, leading to a final chloroform:methanol:water ratio of 1:1:1 (v/v/v). To separate the methanol-water phase containing the water-soluble cellular metabolites from the chloroform phase containing the cellular lipids, the samples were centrifuged for 5 minutes at 5000 rpm and the upper methanol-water phase (8 mL) was carefully removed. Methanol was eliminated from the methanol-water sample using a rotary evaporator, followed by lyophilization. For MR imaging, the lyophilized sample was dissolved in 2 mL PBS to ensure a physiological pH of 7.2 ± 0.1.

Suppl. Method 3: Preparation of tumors for *ex vivo* analysis.

After MR imaging, tumor were removed for further *ex vivo* analysis. For MALDI-2-MSI and immunohistochemistry, tumors were cryo-sectioned in 16 µm tissue slices using a rotary cryomicrotome (Leica Microsystems, Nussloch, Germany). For ^{1}H-NMR spectroscopy of tumor metabolites, extracts of snap-frozen tumors were prepared with the dual-phase extraction method as described above. Tumors were homogenized in a chloroform:methanol:water mixture (2:2:1.8) using a tissue homogenizer (Bertin Technologies, Montigny-le-Bretonneux, France). After centrifugation (-20°C, 5000 rpm, 5 min), the upper methanol-water phase was separated and methanol was removed using a rotary evaporator, followed by lyophilization.

Suppl. Method 4: ^{1}H-NMR spectroscopy.

NMR samples were dissolved in 1.0 mL D_2O (99.98%, Sigma-Aldrich) containing 1 mM maleic acid. Samples were transferred to a 5 mm glass tube for ^{1}H-NMR analysis. Spectra were recorded at 600 MHz using an Agilent DD2 600 spectrometer (Agilent Technologies, Santa Clara, California, USA). ^{1}H-NMR chemical shifts are given relative to tetramethylsilane and are referenced to the solvent signal (HDO, $\delta = 4.66$ ppm). The data were recorded using the manufacturer's software and processed with MestReNova (version 14.2.0, Mestrelab Research, Santiago de Compostela, Spain). An internal standard of 1 mM maleic acid was used for quantitative analysis (singlet occurring at a chemical shift between 6.2 and 6.4 ppm). Concentrations of glucose were quantified from signals at 3.4 ppm, creatine at 2.95 ppm and amide protons as integrals from 6.8 to 9 ppm. For analysis of intratumoral metabolites, concentrations were normalized to the tumor volume, assessed by T2-weighted imaging.

Suppl. Method 5: Immunohistochemical co-staining of GLUT1, GLUT3 and CD3.

After fixation and rehydration, tumor sections were incubated in blocking buffer (2% horse serum and 0.1% fetal calf serum in phosphate-buffered saline, PBS) for 60 minutes at room temperature. Subsequently, the sections were incubated in a humidified chamber overnight at 4°C with primary antibodies against GLUT1 (sheep, 112AP, FabGennix, Frisco, Dallas, USA), GLUT3 (rabbit, 20403-I-AP, proteintech, Manchester, England) and CD3 (allophycocyanin-conjugated, rat, 17A2, BioLegend, San Diego, California, USA), each at a dilution of 1:150 in blocking buffer. After washing the slides three times with blocking buffer, they were incubated for 90 minutes at room temperature with anti-sheep Alexa Fluor 488 (A-11015, Thermo Fisher Scientific, Waltham, Massachusetts, USA) and anti-rabbit Alexa Fluor 568 (A-10042, Thermo Fisher Scientific), each at a dilution of 1:500. Finally, the sections were washed with PBS, stained with 20 µM Hoechst 33342 dye (62249, Thermo Fisher Scientific) for 10 minutes at room temperature, followed by one last washing step with PBS. Microscopy was conducted with an LSM 800 (Carl Zeiss Microscopy, Oberkochen, Germany) and the software ZEN 2.6 (blue edition; Carl Zeiss Microscopy).

Suppl. Method 6: Matrix-assisted laser desorption/ionization mass spectrometry imaging (MALDI-2-MSI).

Conventional mass spectrometry imaging approaches for the quantitative analysis of glucose are hampered by the presence of (near-) isomeric molecules such as inositol. Here, we therefore used a tandem-MS based approach in negative ion mode that utilizes characteristic ring cleavages to identify glucose unambiguously. To achieve signal intensity for deprotonated glucose ions sufficient for tandem-MS directly from tissue we used MALDI-2-MSI and introduce a novel split pixel approach to acquire tandem-MS spectra for glucose and the respective isotopically labeled internal standard in a spatially resolved fashion.

Tissue sections were dried in an evacuated desiccator, vacuum-sealed and stored at -80 °C. At the time of measurement, slides were brought to room temperature in an evacuated desiccator and coated with norharmane matrix for MALDI-2 analysis using a pneumatic spray robot (SunCollect MALDI Sprayer, SunChrome, Germany) with parameters as follows: 3,5 mg/mL norharmane solution in 50:50 (% v/v) acetonitrile:water; total of 20 passages, flow rate: 1st passage 15 µL/min; 2nd passage 20 µL/min; 3rd passage 30 µL/min; all following: 50 µL/min; scan speed: 600 mm/min, line distance 2 mm. Matrix solution was spiked with 4 mg/mL $^{13}C_6$-glucose as internal standard. MALDI-2-MS imaging analysis was conducted on a timsTOF fleX MALDI2 (Bruker Daltonics, Bremen, Germany) in the negative ion mode with microgrid enabled. Acquisition parameters were as follows: Spot size: 14 x 29 µm², step size (x and y) 30 µm, shots per pixel: 150; *m/z* range: 50-500; trigger delay for MALDI-2: 10 µs. Two MALDI-2-MS images were collected from the same region of interest (ROI) of each section with step size of 30 µm in x- and y-direction. For each individual image, a pristine area of 14 x 29 µm² within a 30 x 30 µm² pixel was probed by shifting the ROI by 15 µm in x-direction between measurements. Both runs were collected in tandem-MS mode selecting the de-protonated ion species of glucose at m/z 179 ($^{12}C_6$-glucose) and 185 ($^{13}C_6$-glucose) as precursors, respectively using a selection window of 1 Da. Precursor ions were fragmented using 8 eV of activation energy in low-energy collision induced dissociation (CID). To compare relative intensities between labeled and unlabeled glucose, a characteristic ring cleavage yielding fragment ions at 89.02 for $^{12}C_6$-glucose and 92.05 for $^{13}C_6$-glucose was exploited for unambiguous identification. Images of the respective fragment ions were constructed and analyzed using SCiLS Lab MVS (SCiLS, Bremen, Germany). Quantitative MS images were generated using the SCiLs lab API and a script, written in Python. For this, fragment signal intensity of the unlabeled glucose was normalized using the fragment signal intensity of the internal standard of the same split pixel and multiplied with the respective amount of internal standard applied

per unit area (80.3 pmol/mm²). Because the internal standard is not subject to analyte extraction, the presented quantitative values represent a minimum value for $^{12}C_6$-glucose content in the respective sample. After analysis, matrix was washed off using ethanol. Subsequently, Hematoxylin and eosin (H&E) staining of tumor sections was performed according to standard protocols. The ROIs for further analysis of intratumoral glucose were identified based on bright field microscopy of the H&E-stained samples. Average signal intensity for the ROI of the respective characteristic fragments was generated in SCiLS from the respective tandem-MS data and average quantitative data was generated as described above.

Suppl. Method 7: Dynamic contrast-enhanced MRI (DCE-MRI).

After CEST imaging, dynamic contrast-enhanced (DCE) MRI using Magnevist (Gd-DTPA, 0.3 mmol/kg) was performed to exclude that glucose-weighted CEST results are dominated by perfusion effects. The contrast agent was injected via a tail vein catheter (Klinika Medical GmbH, Usingen, Germany), using a perfusion pump (World Precision Instruments, Sarasota, Florida, USA) at a rate of 240 µL/min. The injection was initiated one minute after starting a fast low-angle shot (FLASH) scan (TR = 24.6 ms, TE = 1.5 ms, 15° flip angle, 1 average, 610 repetitions, 18 x 15 mm^2 FOV, 96 x 96 matrix, acquisition time = 20:00 min:s). Dynamic assessment of contrast enhancement after Magnevist injection was used to derive the tumor perfusion parameter volume transfer constant K_{trans}. Calculation of K_{trans} was performed with an in-house developed software based on the PkModeling extension for 3D Slicer (https://github.com/millerjv/PkModeling), using a three-parameter Tofts model (extended Tofts model) [2] and a population-based arterial input function [3].

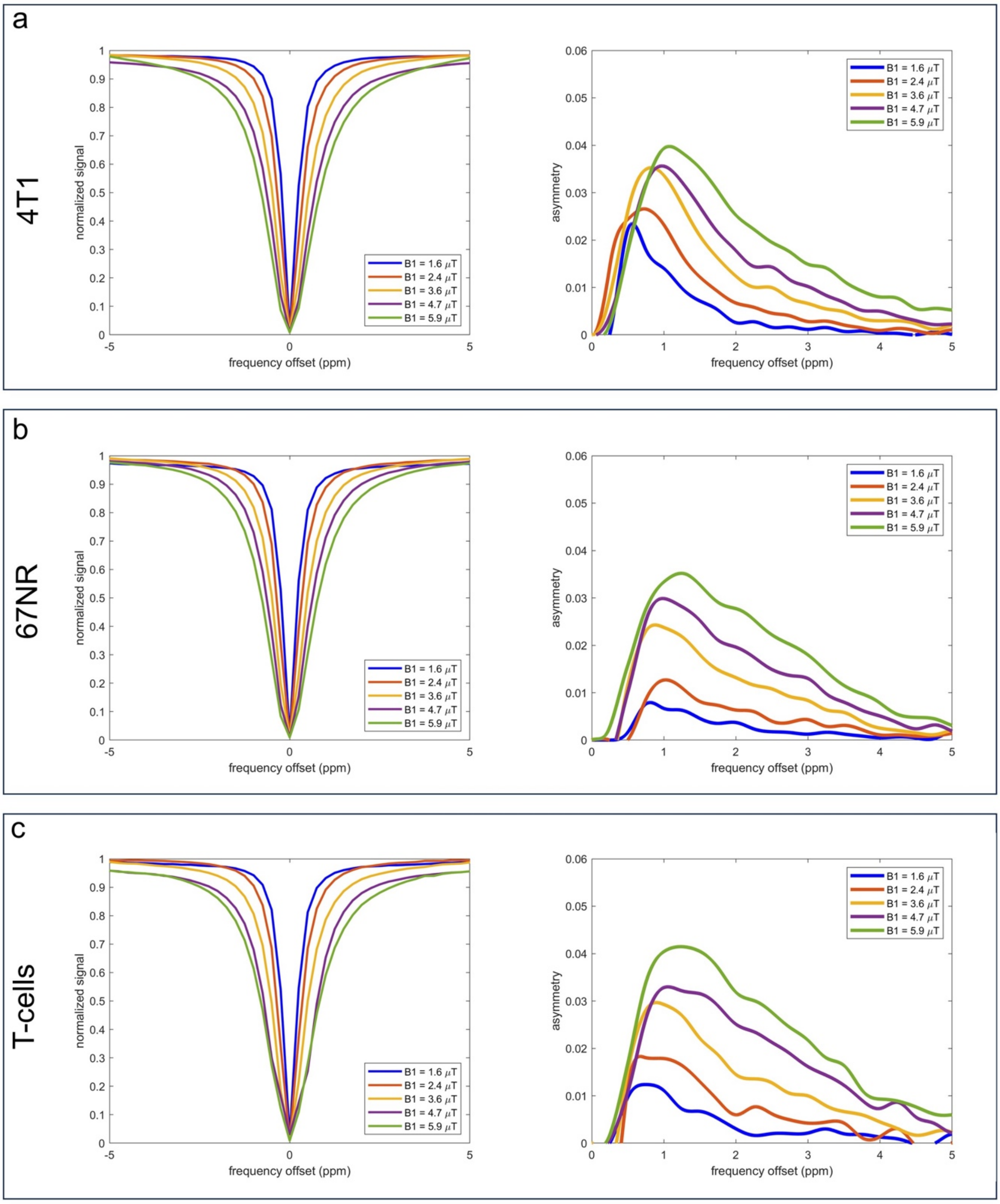

Suppl. Figure 1: Exemplary CEST spectra of *in vitro* cell extracts for different B_1 values.
Exemplary CEST and corresponding asymmetry spectra, acquired for five different B_1 values (1.6, 2.4, 3.6, 4.7 and 5.9 µT) of 4T1 (**a**), 67NR (**b**) and T-cells (**c**). With increasing B_1 values, substantially increased CEST signal was observed, so B_1 = 5.9 µT was used for *in vitro* experiments. However, for following *in vivo* experiments, a lower B_1 value (1.6 µT) was chosen to avoid peak broadening, interference and stronger direct water saturation.

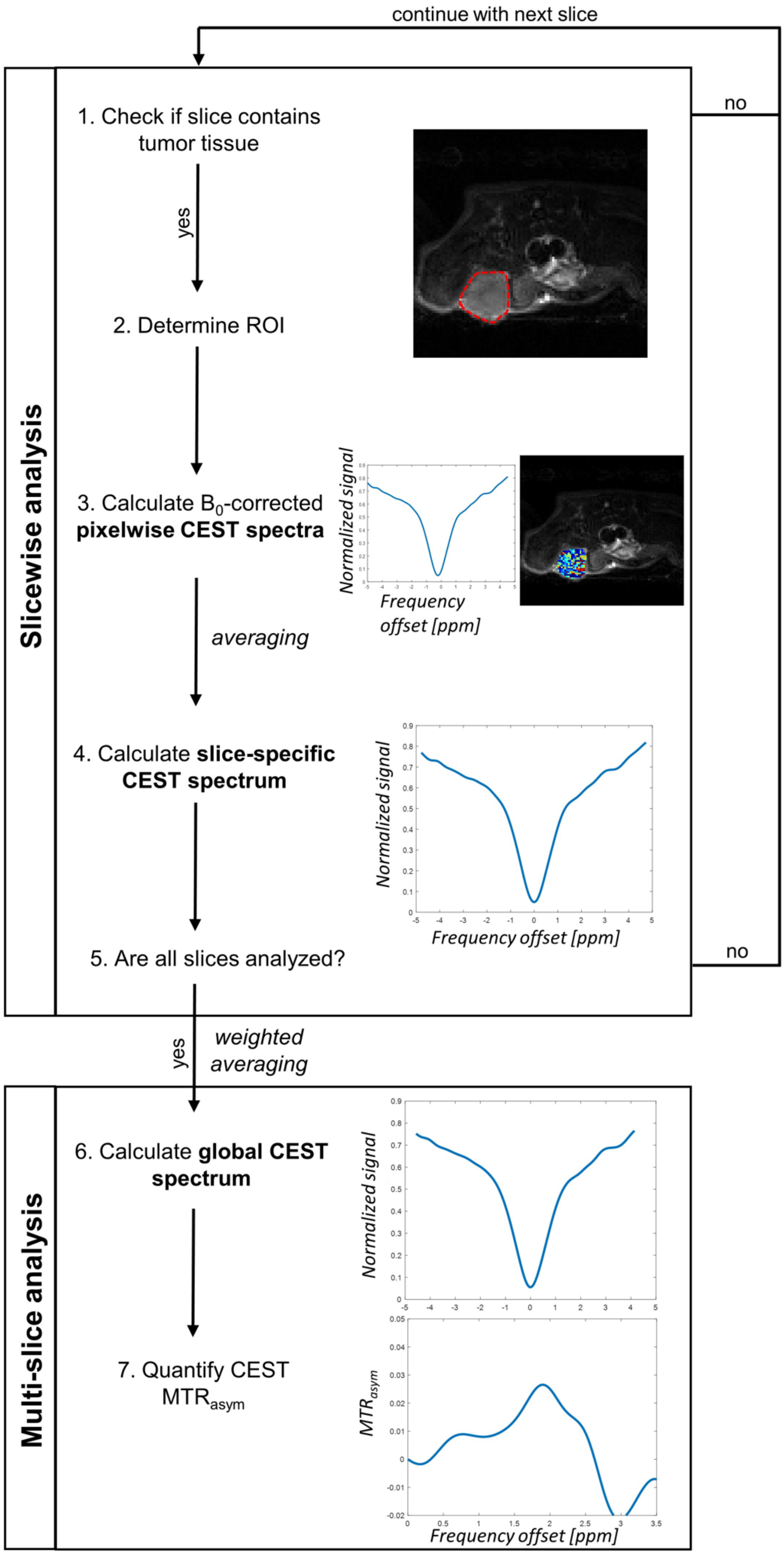

Suppl. Figure 2: Pipeline for *in vivo* CEST-MRI data analysis.
MRI data from multi-slice CEST acquisition were evaluated slicewise. For each slice that contained tumor tissue (1.), a ROI was chosen covering the tumor (2.) and for each pixel within the ROI a CEST spectrum was calculated, B_0-corrected and quantified based on MTR_{asym} values to generate pixelwise CEST contrast maps (3.) Then, pixelwise CEST spectra were averaged to calculate a slice-specific CEST spectrum (4.). Once the slicewise analysis was done for all slices (5.), the slice-specific CEST spectra from each slice were averaged (weighted by the number of pixels representing the tumor size in the slice) to derive one global CEST spectrum (6.). This was then used to calculate and quantify MTR_{asym} contrast at 1.2 – 2.0 ppm (glucose), 2.0 ppm (2ppm peak, creatine) and 3.2 – 3.6 ppm (APT).

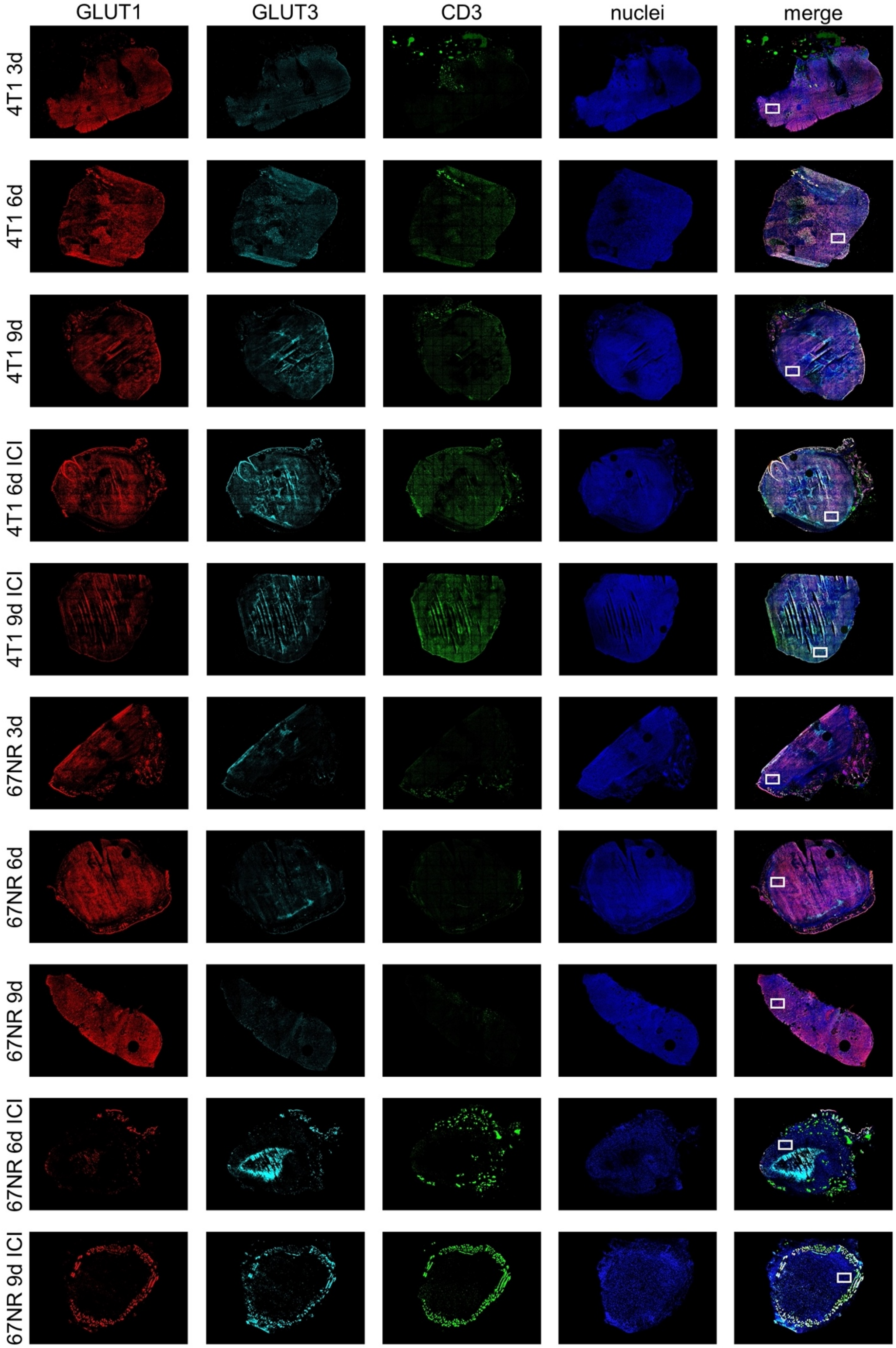

Suppl. Figure 3: Total cross-sections of GLUT1, GLUT3 and CD3 co-stained tumors.
Total cross-sections of 4T1 and 67NR tumors after quadruple immunostaining of GLUT1 (red), GLUT3 (turquoise), CD3 (green) and nuclei (blue) with correlating merged images. Regions of interest that are shown within the main figures Fig. 4a, Fig. 5h and Fig. 6h are indicated with white boxes.

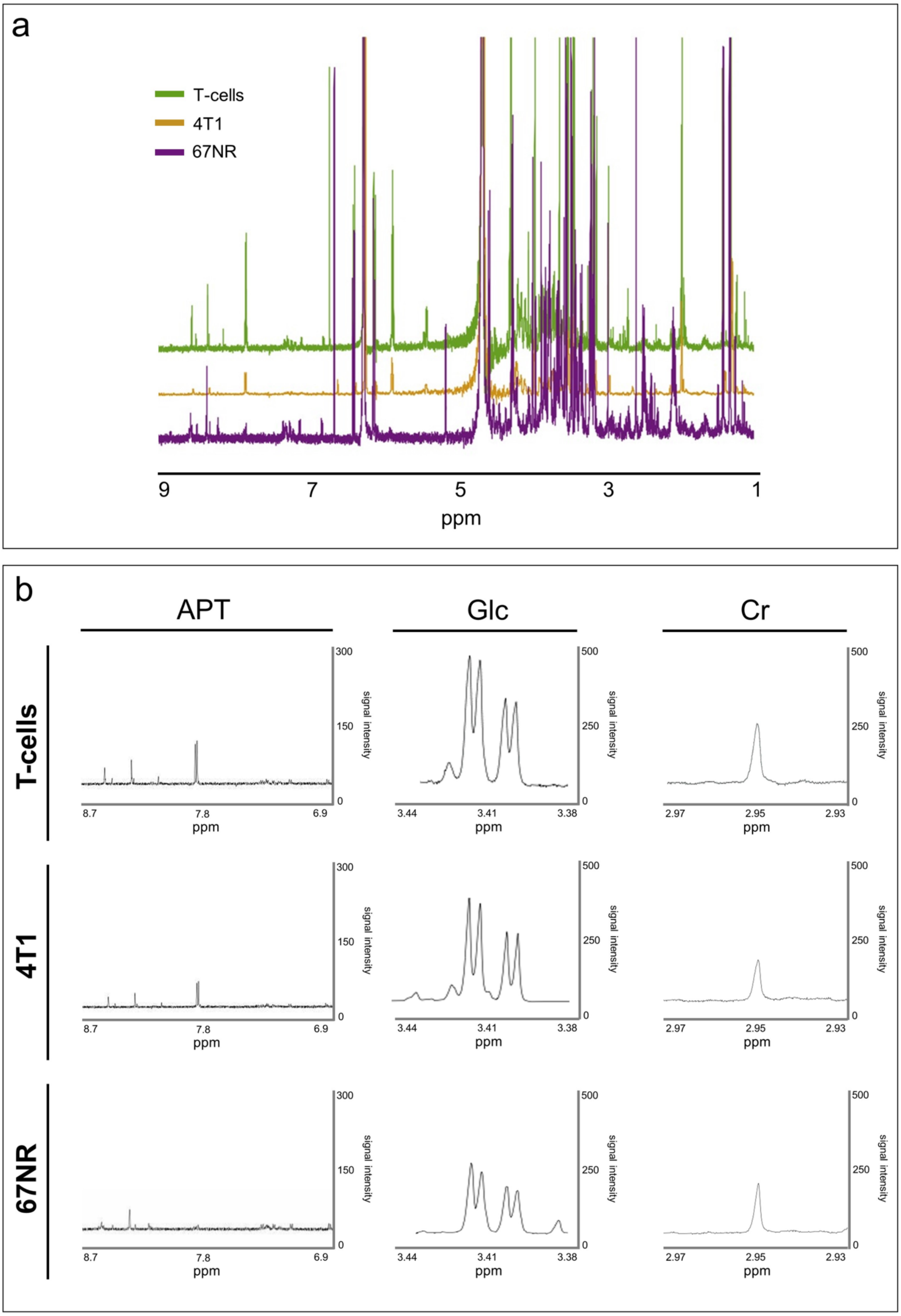

Suppl. Figure 4: Exemplary ^{1}H-NMR spectra of *in vitro* cell extracts.

Exemplary 600 MHz ^{1}H-NMR spectra of 4T1, 67NR and T-cell extracts (**a**), with magnifications for APT, glucose and creatine analysis as described in Suppl. Method 4 (**b**).

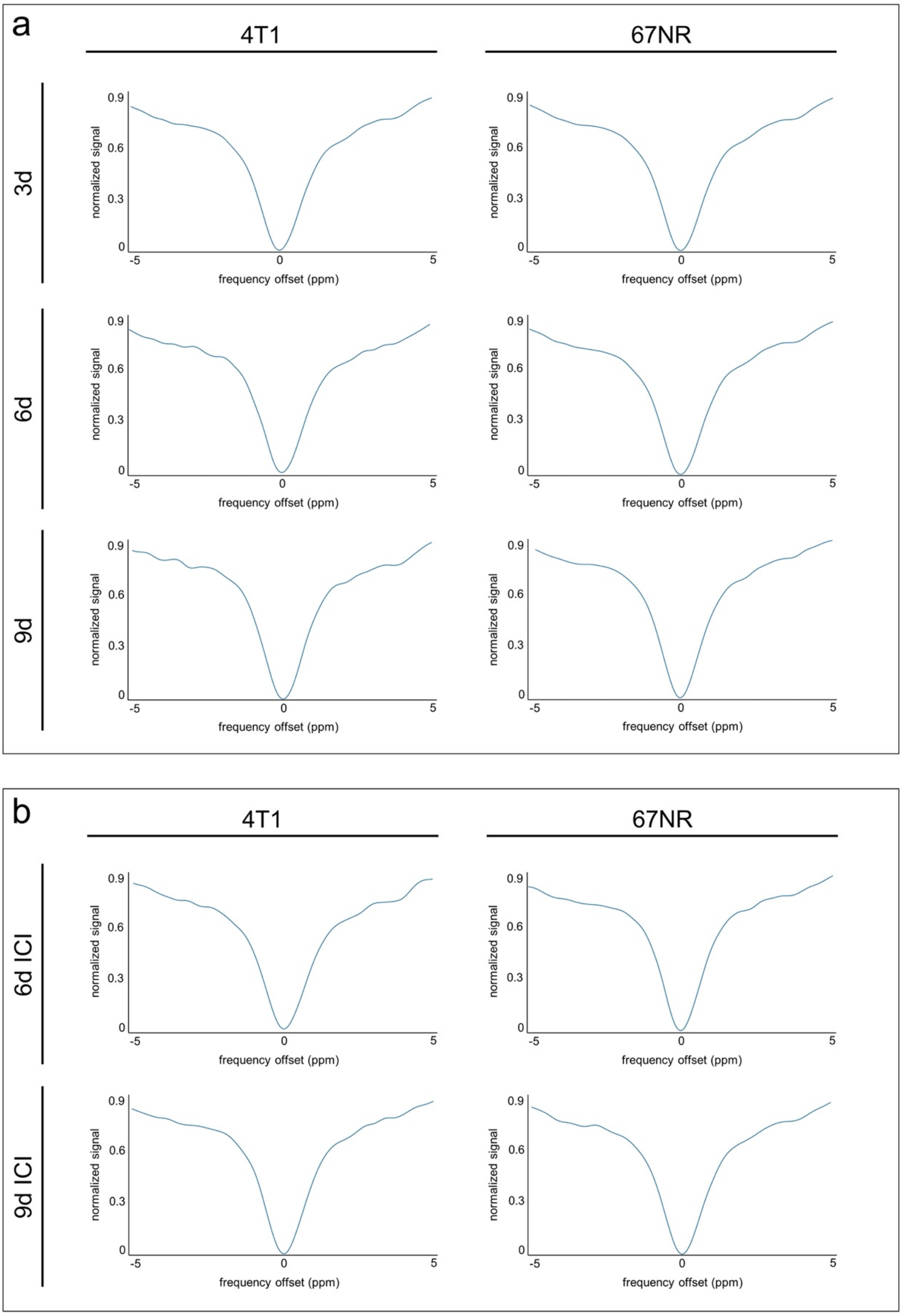

Suppl. Figure 5: Exemplary CEST spectra of *in vivo* CEST-MRI.
Exemplary CEST spectra of 4T1 and 67NR tumors during tumor progression from day three to day nine (**a**) and after immune checkpoint inhibitor (ICI) treatment on day six and day nine (**b**).

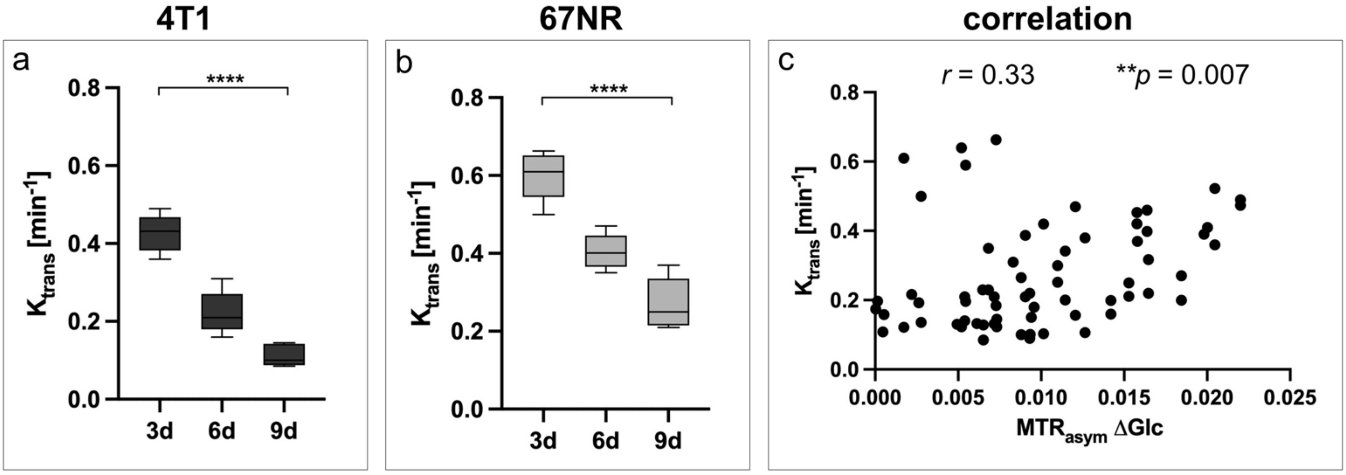

Suppl. Figure 6: Correlation between *in vivo* glucose-weighted CEST-MRI and DCE-MRI.
To exclude that glucose-weighted CEST results are dominated by perfusion effects, CEST-MRI results were compared to the DCE-derived tumor perfusion parameter K_{trans}. DCE-MRI demonstrated decreasing K_{trans} values during progression of both tumor models, with 67NR tumors having higher values at each respective time point (**a**, **b**). Only a low correlation between glucose-weighted CEST contrast and DCE-derived tumor perfusion was found (**c**).

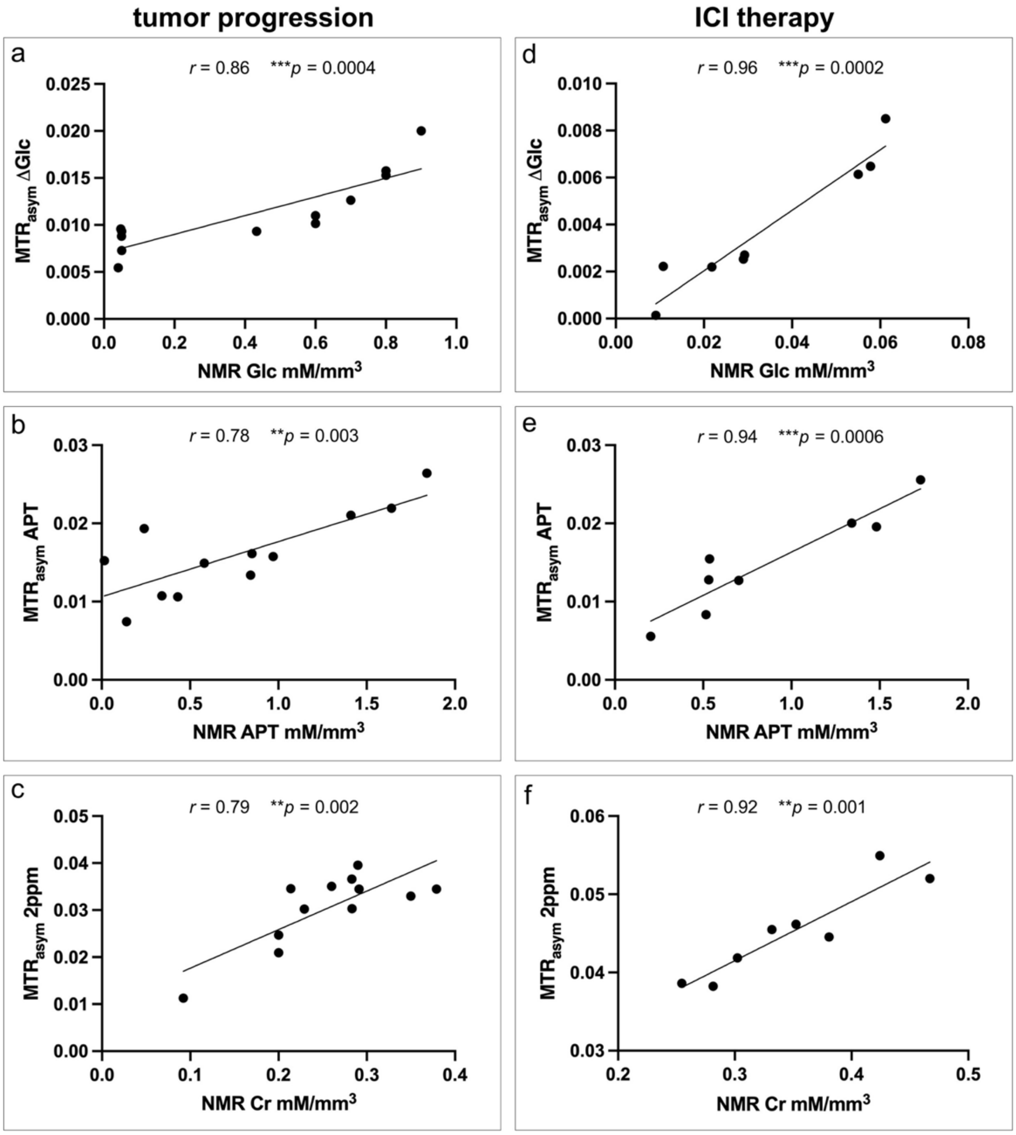

Suppl. Figure 7: Correlation between *in vivo* CEST-MRI and *ex vivo* ^{1}H-NMR spectroscopy.
To validate multiparametric CEST-MRI results, ^{1}H-NMR spectroscopy of homogenized 4T1 and 67NR tumor tissue was performed and concentrations of glucose, amide protons and creatine were quantified. For all analyzed metabolites, a significant positive correlation between CEST-MRI results and metabolite concentrations quantified by ^{1}H-NMR spectroscopy was observed during tumor progression (**a-c**) and after immune checkpoint blockade (ICI, **d-f**).

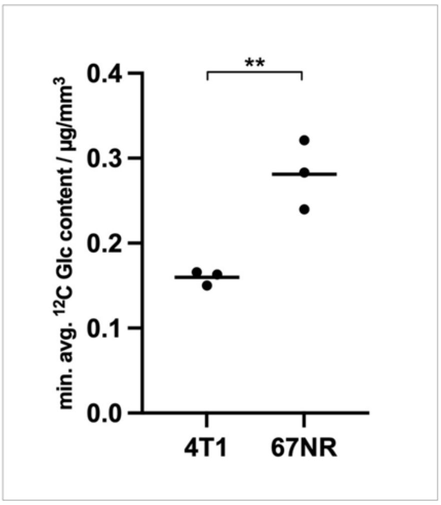

Suppl. Figure 8: Exemplary comparison of intratumoral ^{12}C glucose concentrations assessed by MALDI-2-MSI.
To quantitatively compare the intratumoral concentrations of ^{12}C glucose in one exemplary experimental group, the minimum averaged ^{12}C glucose contents of untreated 4T1 and 67NR tumors nine days after tumor implantation were compared. In line with CEST-MRI and ^{1}H-NMR spectroscopy results, 67NR tumors showed higher intratumoral glucose concentrations.

Suppl. Table 1: Descriptive statistics of *in vitro* cell extracts.
All values are presented as mean ± standard deviation.

**Cell type**	**MTR_{asym} ΔGlc**	**MTR_{asym} APT**	**MTR_{asym} 2ppm**	**NMR c(Glc)** [mM]	**NMR c(APT)** [mM]	**NMR c(Cr)** [mM]
4T1	0.059 ± 0.005	0.0106 ± 0.001	0.0024 ± 0.001	0.039 ± 0.003	0.051 ± 0.007	0.011 ± 0.002
67NR	0.044 ± 0.008	0.0124 ± 0.001	0.0271 ± 0.0005	0.027 ± 0.006	0.048 ± 0.016	0.019 ± 0.001
T-cells	0.078 ± 0.005	0.0192 ± 0.001	0.0391 ± 0.004	0.047 ± 0.003	0.077 ± 0.005	0.031 ± 0.001

Suppl. Table 2: Analysis of statistical significance of *in vitro* cell extracts.

**Compared cell types**	**MTR_{asym} ΔGlc** *p*-value	**MTR_{asym} APT** *p*-value	**MTR_{asym} 2ppm** *p*-value	**NMR c(Glc)** [mM] *p*-value	**NMR c(APT)** [mM] *p*-value	**NMR c(Cr)** [mM] *p*-value
4T1 vs. 67NR	0.04	0.21	0.02	0.05	0.79	0.02
4T1 vs. T-cells	0.01	0.0006	0.02	0.03	0.006	0.0001
67NR vs. T-cells	0.003	0.001	0.04	0.009	0.04	0.0004

Suppl. Table 3: Descriptive statistics of longitudinal *in vivo* study.
All values are presented as mean ± standard deviation.

**Experimental group**	**volume [mm^3]**	**MTR_{asym} ΔGlc**	**MTR_{asym} APT**	**MTR_{asym} 2ppm**	**NMR c(Glc)** [mM/mm^3]	**NMR c(APT)** [mM/mm^3]	**NMR c(Cr)** [mM/mm^3]
4T1 3d	15 ± 5	0.019 ± 0.002	0.024 ± 0.007	0.023 ± 0.012	0.85 ± 0.07	1.34 ± 0.71	0.18 ± 0.14
4T1 6d	49 ± 8	0.011 ± 0.004	0.013 ± 0.004	0.033 ± 0.005	0.49 ± 0.02	0.91 ± 0.08	0.21 ± 0.01
4T1 9d	108 ± 19	0.007 ± 0.002	0.011 ± 0.003	0.037 ± 0.004	0.45 ± 0.07	0.51 ± 0.11	0.28 ± 0.02
67NR 3d	6 ± 3	0.004 ± 0.002	0.007 ± 0.004	0.035 ± 0.004	0.47 ± 0.04	0.24 ± 0.14	0.36 ± 0.02
67NR 6d	21 ± 6	0.011 ± 0.003	0.017 ± 0.003	0.032 ± 0.002	0.65 ± 0.07	0.83 ± 0.83	0.26 ± 0.04
67NR 9d	56 ± 8	0.014 ± 0.003	0.019 ± 0.003	0.024 ± 0.006	0.71 ± 0.14	0.83 ± 1.15	0.24 ± 0.06

Suppl. Table 4: Analysis of statistical significance of longitudinal *in vivo* study.

Experimental group	volume [mm^3] *p*-value	MTR_{asym} ΔGlc *p*-value	MTR_{asym} APT *p*-value	MTR_{asym} 2ppm *p*-value
4T1 3d vs. 4T1 6d	0.0002	0.0007	0.02	0.04
4T1 6d vs. 4T1 9d	< 0.0001	0.11	0.72	0.63
4T1 3d vs. 4T1 9d	< 0.0001	< 0.0001	0.0009	0.0009
67NR 3d vs. 67NR 6d	0.0001	0.02	0.002	0.7
67NR 6d vs. 67NR 9d	< 0.0001	0.26	0.76	0.08
67NR 3d vs. 67NR 9d	< 0.0001	0.0008	0.0004	0.01
4T1 3d vs. 67NR 3d	0.0002	< 0.0001	0.0006	0.03
4T1 6d vs. 67NR 6d	< 0.0001	0.73	0.003	0.64
4T1 9d vs. 67NR 9d	0.0005	0.006	0.002	0.005

Suppl. Table 5: Descriptive statistics of *in vivo* ICI therapy.

All values are presented as mean ± standard deviation.

Experimental group	volume [mm^3]	MTR_{asym} ΔGlc	MTR_{asym} APT	MTR_{asym} 2ppm	NMR c(Glc) [mM/mm^3]	NMR c(APT) [mM/mm^3]	NMR c(Cr) [mM/mm^3]
4T1 6d ICI	69 ± 14	0.005 ± 0.003	0.021 ± 0.006	0.041 ± 0.007	0.056 ± 0.002	1.54 ± 0.28	0.32 ± 0.05
4T1 9d ICI	159 ± 33	0.003 ± 0.002	0.016 ± 0.002	0.048 ± 0.004	0.015 ± 0.009	1.01 ± 0.67	0.38 ± 0.12
67NR 6d ICI	13 ± 4	0.004 ± 0.003	0.013 ± 0.005	0.042 ± 0.005	0.045 ± 0.023	0.52 ± 0.01	0.29 ± 0.05
67NR 9d ICI	19 ± 5	0.003 ± 0.001	0.011 ± 0.003	0.043 ± 0.012	0.022 ± 0.011	0.45 ± 0.35	0.41 ± 0.03

Suppl. Table 6: Analysis of statistical significance of *in vivo* ICI therapy.

Experimental group	volume [mm^3] *p*-value	MTR_{asym} ΔGlc *p*-value	MTR_{asym} APT *p*-value	MTR_{asym} 2ppm *p*-value
4T1 6d ctrl vs. ICI	0.01	0.02	0.02	0.05
4T1 9d ctrl vs. ICI	0.01	0.02	0.01	0.004
67NR 6d ctrl vs. ICI	0.02	0.01	0.16	0.01
67NR 9d ctrl vs. ICI	< 0.0001	0.0002	0.007	0.02

References

1. Petersen B, Wolf M, Austermann J, van Lent P, Foell D, Ahlmann M, et al. The alarmin Mrp8/14 as regulator of the adaptive immune response during allergic contact dermatitis. EMBO J. 2013;32:100–11. https://doi.org/10.1038/emboj.2012.309.

2. Sourbron SP, Buckley DL. On the scope and interpretation of the Tofts models for DCE-MRI. Magn Reson Med. 2011;66:735–45. https://doi.org/10.1002/mrm.22861.

3. Theis D, Keil B, Heverhagen JT, Béhé M, Klose K-J, Fiebich M. [Determination of the arterial input function in mouse-models using clinical MRI]. Z Med Phys. 2008;18:85–90. https://doi.org/10.1016/j.zemedi.2007.12.002.
